# 5F-Cumyl-PINACA in ‘e-liquids’ for electronic cigarettes: comprehensive characterization of a new type of synthetic cannabinoid in a trendy product including investigations on the in vitro and in vivo phase I metabolism of 5F-Cumyl-PINACA and its non-fluorinated analog Cumyl-PINACA

**DOI:** 10.1007/s11419-018-0451-8

**Published:** 2018-11-13

**Authors:** Verena Angerer, Florian Franz, Bjoern Moosmann, Philippe Bisel, Volker Auwärter

**Affiliations:** 1 Institute of Forensic Medicine, Forensic Toxicology, Medical Center–University of Freiburg, Albertstr. 9, 79104 Freiburg, Germany; 2 Faculty of Medicine, University of Freiburg, Breisacher Str. 153, 79110 Freiburg, Germany; 3 Hermann Staudinger Graduate School, University of Freiburg, Hebelstr. 27, 79104 Freiburg, Germany; 4 Institute of Forensic Medicine, Forensic Toxicology, Kantonsspital St. Gallen, Rorschacher Str. 95, 9007 St. Gallen, Switzerland; 5 Institute for Pharmaceutical Sciences, University of Freiburg, Albertstr. 25, 79104 Freiburg, Germany

**Keywords:** 5F-Cumyl-PINACA, Cumyl-PINACA, In vivo and in vitro metabolism, E-liquids, cAMP assay, LC–MS/MS

## Abstract

**Purpose:**

In recent years e-liquids used in electronic cigarettes have become an attractive alternative to smoking tobacco. A new trend is the use of e-liquids containing synthetic cannabinoids (SCs) instead of smoking cannabis or herbal mixtures laced with SCs. In the frame of a systematic monitoring of the online market of ‘legal high’ products, e-liquids from online retailers who also sell herbal blends were bought.

**Methods:**

The products were analyzed by gas chromatography-mass spectrometry. In some of the e-liquids an unknown compound was detected which was identified as the SC 5F-Cumyl-PINACA (1-(5-fluoropentyl)-*N*-(2-phenylpropan-2-yl)-1*H*-indazole-3-carboxamide) by nuclear magnetic resonance analysis. To investigate the phase I metabolism of this new class of compounds, 5F-Cumyl-PINACA and its non-fluorinated analog Cumyl-PINACA were incubated with pooled human liver microsomes (pHLM). Cumyl-PINACA was additionally ingested orally (0.6 mg) by a volunteer in a controlled self-experiment. To assess the relative potency of Cumyl-PINACA a set of SCs were characterized using a cAMP assay.

**Results:**

Metabolism of 5F-Cumyl-PINACA and Cumyl-PINACA showed similarities with AM-2201 and JWH-018. The main metabolites were formed by hydroxylation at the *N*-pentyl side chain. The main metabolites detected in the volunteer’s urine sample were the same as in the pHLM assay. All SCs tested with the cAMP assay were full agonists at the CB_1_ receptor. Cumyl-PINACA was the most potent SC among the tested compounds and showed an EC_50_ value of 0.06 nM.

**Conclusions:**

The increasing popularity of e-liquids particularly among young people, and the extreme potency of the added SCs, pose a serious threat to public health. To our knowledge, this is the first report describing the tentative identification of human in vivo metabolites of Cumyl-PINACA and 5F-Cumyl-PINACA.

**Electronic supplementary material:**

The online version of this article (10.1007/s11419-018-0451-8) contains supplementary material, which is available to authorized users.

## Introduction

Synthetic cannabinoids have been sold for over a decade as a ‘legal’ alternative to cannabis. To date, these drugs were predominately offered as herbal blends (e.g., ‘Spice’, ‘K2’) with the compounds being sprayed onto dried plant material [1–3]. In contrast to cannabis, intoxications with synthetic cannabinoids can lead to life-threatening conditions [4–6] and the number of patients who were delivered to emergency rooms after consumption of synthetic cannabinoids increased during the last years [7, 8].

One of the first substances identified in herbal mixtures was JWH-018, which belongs to the group of the naphthoylindoles, often referred to as ‘first generation’ synthetic cannabinoids [9]. This paper reports on a new phenomenon first observed in 2015—e-liquids laced with synthetic cannabinoids. In 2016 e-liquids containing MDMB-FUBINACA were identified in the USA [10].

Electronic cigarettes have become increasingly popular for supporting cessation of tobacco smoking and among young people as ‘e-hookahs’ [11–13]. The heating solution (‘liquid’) used for e-cigarettes is typically consisting of propylene glycol or glycerol, nicotine and flavors [14]. Over the last years, some online retailers selling ‘herbal blends’ added e-liquids to their product range. In abstinence control settings, consumption of synthetic cannabinoid containing e-liquids might go undetected due to insufficient testing methods and the relatively unsuspicious form of application.

In the frame of the EU-projects ‘SPICE’, ‘SPICE II Plus’ and ‘SPICE Profiling’ which included a systematic monitoring of the online market of ‘legal highs’, 21 e-liquids were bought from online retailers who also sold herbal blends.

In three of the e-liquids (named as ‘c-liquids’) bought in September 2014, an unknown synthetic cannabinoid was detected. The structure of this unknown substance was identified using nuclear magnetic resonance (NMR) spectroscopy. The unknown compound was identified as 5F-Cumyl-PINACA (1-(5-fluoropentyl)-*N*-(2-phenylpropan-2-yl)-1*H*-indazole-3-carboxamide) which was reported to the European information system and database of new drugs (EDND) in the meantime by the Swedish National Laboratory of Forensic Science. In July 2013, New Zealand passed a ‘Psychoactive Substances Act’ which allowed the sale of approved products containing new psychoactive substances. Only licensed manufacturers were allowed to sell these products, and they had to prove the relatively low risk of these substances beforehand by preclinical and clinical trials. Until all tests were done, there was an interim regime allowing to trade these products while they were tested [15]. In May 2014, an amendment of the act ended this interim period. Between July 2013 and May 2014, several synthetic cannabinoids called ‘SGT’ compounds (probably short for ‘Stargate’) were sold by various manufacturers. The 5F-Cumyl-PINACA was among these compounds and sold as SGT-25 [16]. Since the amendment of the act in May 2014, no new psychoactive substance was approved for sale [17].

For investigation of the phase I metabolism of the new cumyl derivatives, 5F-Cumyl-PINACA and its non-fluorinated analog Cumyl-PINACA (sold as SGT-24 [15], 1-pentyl-*N*-(2-phenylpropan-2-yl)-1*H*-indazole-3-carboxamide) were incubated with pooled human liver microsomes (pHLM). When 5F-Cumyl-PINACA was identified in e-liquids at the Forensic Toxicology Laboratory in Freiburg, the Slovenian National Forensic Laboratory reported on several other cumyl derivatives via the EDND and kindly provided highly pure reference standard of the non-fluorinated analog Cumyl-PINACA. It is for this reason that Cumyl-PINACA and not its fluorinated analog was used for further experiments. A controlled self-experiment with one volunteer ingesting a low dose of Cumyl-PINACA orally was performed to investigate its in vivo phase I metabolism.

Because the cumyl derivatives are a relatively new group of synthetic cannabinoids, and no information about their binding affinities to the cannabinoid receptor 1 (CB_1_) was available until 2017, Cumyl-PINACA was characterized together with a selection of other synthetic cannabinoids using a cAMP biosensor assay with CB_1_ as target.

Since 2015, a number of cumyl derivatives was identified on the legal high market [18–20], leading to the necessity of developing methods to detect metabolites of cumyl derivatives in urine samples. The metabolism of a few cumyl derivatives has already been investigated in vitro in the last years [21–24]. Nevertheless, the paper presented here provides additional data on a self-experiment with ingestion of Cumyl-PINACA. This enabled comparison of in vitro and in vivo metabolites and of some basic kinetic data for Cumyl-PINACA in serum and its metabolites in urine samples.

## Materials and methods

### Chemicals and reagents

Acetonitrile (LC–MS grade), ammonium formate 10 M (99.995%), ethanol absolute, ethyl acetate p.a., methanol (Chromasolv^TM^), potassium hydroxide (purris, p.a., ≥ 86% (T) pellets) and superoxide dismutase (SOD) (≥ 3,000 units/mg protein from bovine erythrocytes, 1 mg/mL solution, dissolved in 0.5 M potassium phosphate buffer pH 7.5) were purchased from Sigma-Aldrich (Steinheim, Germany); sodium carbonate and *n*-hexane (Lichrosolv^®^) from Merck (Darmstadt, Germany); formic acid (98–100%, p.a.) from Applichem (Darmstadt, Germany); potassium hydrogen phosphate (≥ 99%, p.a.) and sodium hydrogen carbonate from Carl Roth (Karlsruhe, Germany); β-glucuronidase (*E. coli* K 12) from Roche Diagnostics (Mannheim, Germany); pHLMs (50 donors, 20 mg/mL protein in 250 mM sucrose), NADPH regenerating solutions A/B (reductase activity 0.43 µmol/min × mL), and 0.5 M potassium phosphate buffer (pH 7.5) from Corning (Corning, NY, USA). NADPH regenerating solution A consisted of 26 mM NADP^+^, 66 mM glucose-6-phosphate, and 66 mM MgCl_2_ in water. NADPH regenerating solution B consisted of 40 U/mL glucose-6-phosphate dehydrogenase in 5 mM sodium citrate. Deionized water was prepared using a Medica^®^ Pro deionizer from ELGA (Celle, Germany).

Cumyl-PINACA (purity > 98%) was kindly provided by the Slovenian National Forensic Laboratory (Ljubljana, Slovenia). The 5F-Cumyl-PINACA, ADBICA-*d*_9_, AKB48-*d*_9_, JWH-007-*d*_9_, JWH-398-*d*_9_, MAM-2201-*d*_5_, PB-22-*d*_9_, RCS-4*d*_9_, UR-144-*d*_5_ and XLR-11-*d*_5_ were purchased from Cayman Chemical (Ann Arbor, MI, USA); JWH-015-*d*_7_, JWH-018-*d*_11_, JWH-081-*d*_9_, JWH-122-*d*_9_, JWH-200-*d*_5_, JWH-210-*d*_9_ and JWH-250-*d*_5_ from LGC Standards (Wesel, Germany); JWH-018 and JWH-073-*d*_9_ from Chiron AS (Trondheim, Norway). AB-CHMINACA, AB-FUBINACA, AB-PINACA, AM-2201, EG-018, MDMB-CHMICA and THJ-2201 were bought as ‘research chemical’ via the Internet; identity and purity (≥ 95%) were determined using gas chromatography–mass spectrometry (GC–MS) and liquid chromatography–tandem mass spectrometry (LC–MS/MS).

The cAMP biosensor assay with CB_1_ as target was conducted by DiscoveRx (Fremont, CA, USA) with the substances AB-CHMINACA, AB-FUBINACA, AB-PINACA, AM-2201, Cumyl-PINACA, EG-018, JWH-018, MDMB-CHMICA and THJ-2201. Each compound was tested in duplicates; to induce response, 20 µM forskolin was used.

For LC–MS/MS analysis, mobile phase A consisted of 1% acetonitrile, 0.1% formic acid, and 2 mM ammonium formate in water, while mobile phase B consisted of acetonitrile with 0.1% formic acid, and 2 mM ammonium formate.

### Instrumentation

#### GC–MS analysis

For GC–MS analysis, a 6890 series chromatography system combined with a 5973-series mass selective detector, a 7683 B series injector and Chemstation G1701GA version D.03.00.611 (Agilent, Waldbronn, Germany) was used. The method parameters were as described elsewhere [25]. Briefly they were: injection port temperature 270 °C, carrier gas helium, flow rate 1 mL/min, and oven temperature 100 °C for 3 min, ramped to 310 °C with 30 °C/min and held for 10 min. Electron ionization (EI) mode with 70 eV ionization energy and scan mode from *m/z* 40–550 were applied. The obtained mass spectra were compared to commonly used EI–MS spectra libraries (Cayman Chemical, Scientific Working Group for the Analysis of Seized Drugs (SWGDRUG), National Institute of Standards and Technology (NIST), Wiley and Maurer-Pfleger-Weber (MPW)), to the EDND of the European Monitoring Center for Drugs and Drug Addiction (EMCDDA) and to an in-house library of previously identified synthetic cannabinoids.

#### NMR analysis

NMR spectra of the unknown compound found in three of the e-liquids (called ‘c-liquids’ by some vendors) were recorded in CDCl_3_ at room temperature with a DRX 400 (Bruker Physik AG, Ettlingen, Germany). Chemical shifts are reported in ppm relative to CHCl_3_ (^1^H: *d* = 7.27) and CDCl_3_ (^13^C: *d* = 77.23) as internal standards. Compounds were fully characterized by 1D-^1^H as well as ^13^C NMR at 400 MHz and 100 MHz, respectively. Selective 1D-TOCSY (total correlation spectroscopy), 1D-ROESY (rotation frame nuclear Overhauser effect spectroscopy) as well as 2D-^1^H/^13^C HSQC (heteronuclear single quantum coherence), ^1^H/^1^H COSY (correlation spectroscopy) and ^1^H/^13^C HMBC (heteronuclear multiple-bond correlation spectroscopy) spectra were recorded.

#### LC–MS/MS analysis of serum samples

For analysis of the serum samples, a routinely used LC–MS/MS screening method, currently covering 94 compounds with at least two ion transitions for each analyte and one ion transition for each internal standard in positive scheduled multiple reaction monitoring (+sMRM) was applied [26]. Briefly, a QTRAP™ 4000 triple quadrupole linear ion trap instrument (Sciex, Darmstadt, Germany) equipped with a TurbolonSpray^®^ interface and coupled to a Shimadzu Prominence HPLC system consisting of two LC-20 AD SP isocratic pumps, SIL 20 AC autosampler, CTO-20A controller (Shimadzu, Duisburg, Germany) and Analyst^®^ software version 1.6.2 were used (Sciex). Chromatographic separation was performed on a Kinetex^®^ C18 column (2.6 µm, 100 Å, 100 × 2.1 mm; Phenomenex, Aschaffenburg, Germany) applying gradient elution as follows: starting concentration of 20% mobile phase B was held for 1 min, then linearly increased to 60% B for 1.5 min, further increased to 65% B for 1.5 min, held for 1.5 min, further increased to 90% B for 2.5 min, held for 2.0 min, decreased to starting conditions of 20% B for 0.1 min and held for 1.9 min for re-equilibration. The total flow rate was 0.5 mL/min. The autosampler and the column oven temperatures were set to 10 and 40 °C, respectively. The monitored ion transitions used for Cumyl-PINACA were *m/z* 350→215 and 350→232.

#### LC–MS/MS experiments for metabolite identification (pHLM and urine samples)

The LC–MS/MS system used for the analysis of the urine and the pHLM assay samples consisted of a Nexera X2 UHPLC (Shimadzu) coupled to a QTRAP™ 5500 triple quadrupole linear ion trap instrument (SCIEX) equipped with the TurboIonSpray^®^ probe. Chromatographic separation was performed on the Kinetex^®^ C18 column (2.6 µm, 100 Å, 100 × 2.1 mm) applying gradient elution as follows: starting concentration of 5% mobile phase B was held for 0.5 min, then linearly increased to 35% B for 0.5 min, further increased to 50% B for 3.0 min, further increased to 90% B for 4.0 min, held for 2.0 min, decreased to starting conditions of 5% for 0.5 min and held for 2.5 min for re-equilibration. The total flow rate was 0.4 mL/min. The autosampler and the column oven temperatures were set to 10 and 40 °C, respectively.

The MRM ion transitions of the parent compounds were optimized carefully in positive ionization mode (see Supplementary Tables S1 and S2). Enhanced product ion (EPI) scan experiments were performed using hypothetical masses of anticipated phase I metabolites, and the obtained spectra were compared to the EPI spectrum of the respective parent compound. In order to detect unexpected metabolites not covered by the EPI scan approach, precursor ion scan experiments for the characteristic fragment ion at *m/z* 145 and for the *m/z* of its modified analogs 161 (monohydroxylation), 177 (dihydroxylation) and 179 (dihydrodiol) were conducted. The relative abundances of the characterized metabolites in the urine samples obtained during the self-administration study were compared using an MRM method comprising the most abundant ion transitions of each metabolite (see Supplementary Fig. S1 and Table S3).

### Study design

#### Self-administration study

To investigate the in vivo phase I metabolism of Cumyl-PINACA, one of the authors (28 years, 75 kg, Caucasian male) ingested 0.6 mg Cumyl-PINACA orally. In addition to blank samples taken prior to ingestion, serum and urine samples were collected repeatedly over periods of 17 and 31 h after ingestion, respectively. All samples were stored at − 20 °C until analysis. In Germany, approval by an ethics committee is not required for scientific self-experiments.

#### Authentic urine sample

The analysis of in vivo metabolism of 5F-Cumyl-PINACA in an authentic urine sample was conducted in accordance with the client’s inquest. Hence, no approval by an ethics committee was required.

#### Control samples

Blank serum and urine samples were provided by healthy volunteers and were tested for the absence of synthetic cannabinoids and their metabolites prior to use.

### Sample treatments

#### E-liquids

A 1-mL volume of the e-liquids bought during the monitoring of the online market was extracted by adding 1 mL of methanol and 1 mL *n*-hexane using an overhead shaker for 5 min at lowest rotation speed (Reax 2; Heidolph, Schwabach, Germany). Subsequently, samples were centrifuged at 2860 × *g* for 5 min (Heraeus Megafuge 1.0; Thermo Scientific, Schwerte, Germany). Ten microliters of the supernatant were evaporated to dryness at 40 °C under a gentle stream of nitrogen. Prior to analysis by the GC–MS system (injection volume 1 µL), the samples were reconstituted in 100 µL of dry ethyl acetate.

For NMR analysis, 1 mL of an e-liquid was extracted five times using *n*-hexane/ethyl acetate (99:1, v/v). The combined supernatants were evaporated to dryness and resulted in 4 mg of a raw extract containing 5F-Cumyl-PINACA, which was used for structure elucidation by NMR spectroscopy.

#### Pooled human liver microsome assay

To investigate the tentative phase I metabolism of Cumyl-PINACA and 5F-Cumyl-PINACA (extracted from e-liquids), in vitro experiments with pHLM were performed in triplicates. A 0.5-µL volume of a substrate solution (1 ng/mL in acetonitrile) was added to 49.5 µL of a reaction mixture consisting of 2.5 µL pHLM, 2.5 µL NADPH regenerating solution A, 0.5 µL NADPH regenerating solution B, 5.0 µL SOD, 10 µL 0.5 M phosphate buffer, and 29 µL deionized water. Incubation was performed for 60 min at 37 °C. The reaction was terminated by the addition of ice-cold acetonitrile, and the supernatant was analyzed after 1:10 dilution with mobile phase A/B (50:50, v/v). Blank pHLM samples were processed in the same way serving as negative controls.

#### Serum samples

A 1-mL aliquot of a serum sample was spiked with 10 µL of an internal standard solution mixture (25 ng/mL each, ADBICA-*d*_9_, AKB48-*d*_9_, JWH-007-*d*_9_, JWH-015-*d*_7_, JWH-018-*d*_11_, JWH-073-*d*_9_, JWH-081-*d*_9_, JWH-122-*d*_9_, JWH-200-*d*_5_, JWH-210-*d*_9_, JWH-250-*d*_5_, JWH-398-*d*_9_, MAM-2201-*d*_5_, PB-22-*d*_9_, RCS-4-*d*_9_, UR-144-*d*_5_, XLR-11-*d*_5_). A 0.5-mL volume carbonate buffer (pH 10) and 1.5 mL of extraction mixture 1 (*n*-hexane/ ethyl acetate, 99:1, v/v) were added. After gentle mixing for 5 min, the sample was centrifuged and 1 mL of the supernatant was transferred to an HPLC vial. Subsequently, 1.5 mL of extraction mixture 2 (*n-*hexane/ethyl acetate, 80:20, v/v) was added to the residue, and the mixing and centrifugation steps were repeated. A 1-mL volume of the supernatant was added to the same HPLC vial, and after evaporation under a gentle stream of nitrogen at 40 °C and reconstitution in 100 µL of mobile phase (A/B, 80:20, v/v), the sample was analyzed using LC–MS/MS.

#### Urine samples

In order to cleave conjugates, 0.5 mL of an urine sample was incubated at 45 °C for 1 h after addition of 0.5 mL phosphate buffer (pH 6) and 30 µL β-glucuronidase. In a next step, 1.5 mL acetonitrile and 0.5 mL ammonium formate (10 M) were added. After shaking and centrifugation, 1 mL of the acetonitrile phase was transferred into a glass vial and evaporated to dryness under a gentle nitrogen stream at 40 °C. The residue was reconstituted in 200 µL mobile phase A/B (50:50, v/v) directly before LC–MS/MS analysis. Blank urine samples were prepared serving as negative controls.

## Results and discussion

### Qualitative analysis of e-liquids

Twenty-one e-liquids offered via the Internet and bought between May 2014 and June 2015 were analyzed; ten of them (47%) contained one or two synthetic cannabinoids. In three e-liquids, an unknown substance was detected (GC–MS spectrum of the unknown substance see Supplementary Fig. S2) which could be identified as 5F-Cumyl-PINACA (SGT-25); four e-liquids contained 5F-AKB48 (5F-APINACA), two contained AB-CHMINACA as the active ingredient and one contained AB-FUBINACA and AB-PINACA.

### Structural elucidation of the unknown compound

**Fig. 1:**
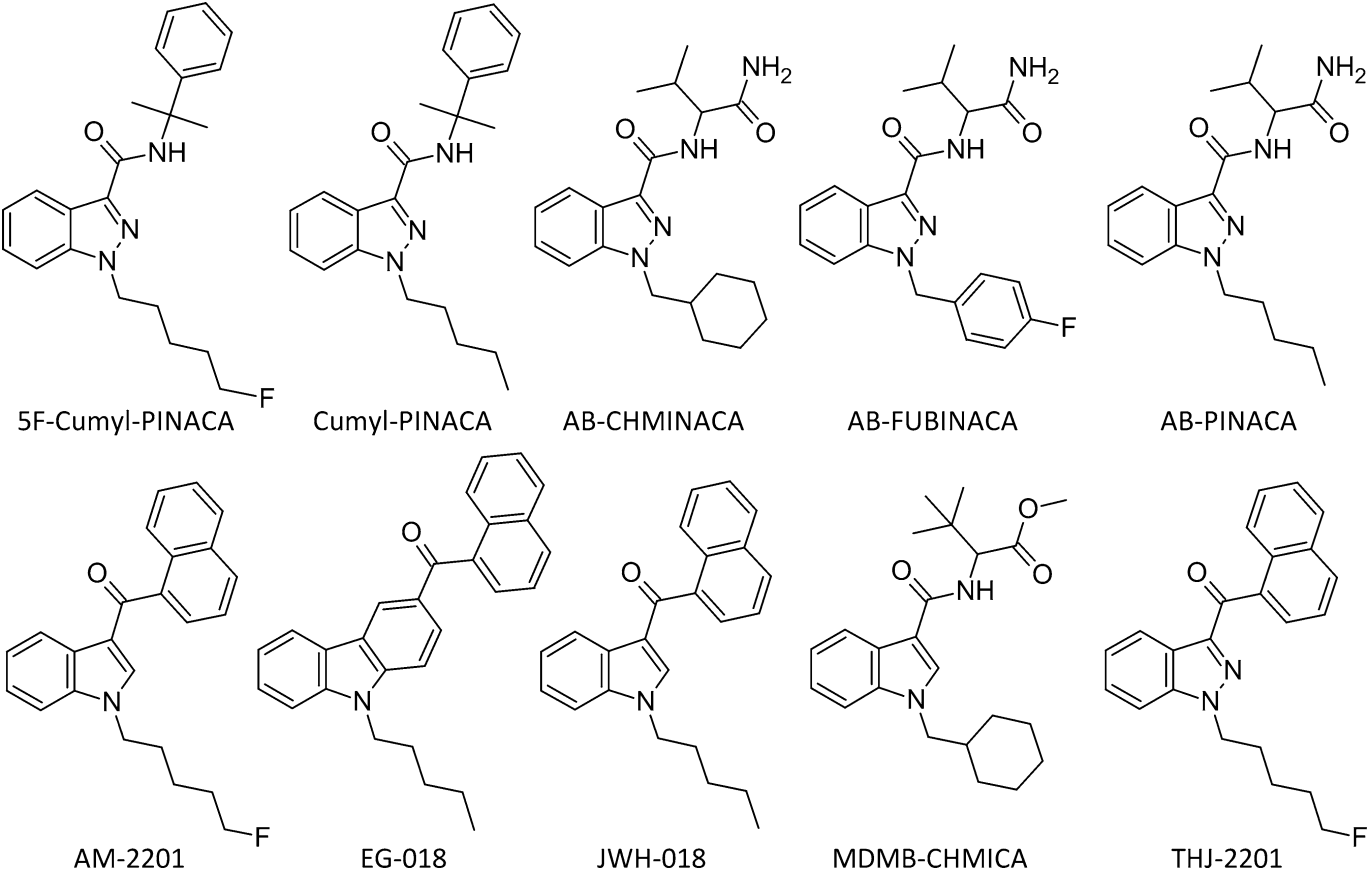
Chemical structures of 5F-Cumyl-PINACA and the other synthetic cannabinoids tested by the cAMP biosensor assay

By ^1^H- and ^13^C-NMR experiments (see Supplementary Fig. S3 and Table S4), the structure of the unknown compound was resolved as 5F-Cumyl-PINACA (1-(5-fluoropentyl)-*N*-(2-phenylpropan-2-yl)-1*H*-indazole-3-carboxamide) (see Fig. 1). Following up the study, these data were additionally verified by comparison to a commercial reference standard.

### cAMP biosensor assay

Cumyl-PINACA has an indazole core and not an indole core (like, e.g., JWH-018 with an EC_50_ of 1.13 nM), which often leads to a relatively low potency as seen in the example of AM-2201 (indole core, EC_50_ = 0.45 nM) and THJ-2201 (indazole core, EC_50_ = 1.68 nM). Nevertheless, it seems that the cumyl moiety significantly increases the potency of a synthetic cannabinoid (Cumyl-PINACA EC_50_ = 0.06 nM). As compared to all the other tested substances, Cumyl-PINACA was the most potent substance in this assay. Except EG-018, all substances tested in this experiment showed full agonistic activity at the CB_1_ receptor. All results of the biosensor assay are listed in Table 1 and all structural formulae are shown in Fig. 1. The reference standard of 5F-Cumyl-PINACA was not available for comparison at the time when this study was conducted.

**Table 1:** Results of the cAMP biosensor assay

Compound name	Assay format	EC_50_ (nM)	Curve top	Maximum response (compared to CP-55,940)^a^
AB-CHMINACA	Agonist	0.28	94.8	94.827
AB-FUBINACA	Agonist	0.89	97.4	97.82
AB-PINACA	Agonist	1.74	92.3	95.254
AM-2201	Agonist	0.45	103	101.41
Cumyl-PINACA	Agonist	0.06	93.7	92.86
EG-018	Agonist	40.7	74.3	71.826
JWH-018	Agonist	1.13	97.6	97.392
MDMB-CHMICA	Agonist	0.14	94.8	94.57
THJ-2201	Agonist	1.68	95.6	91.92

^a^Control ligand CP-55,940

Several studies were conducted since 2015 to evaluate the relative potency of cumyl derivatives as compared to other synthetic cannabinoids. Longworth et al. [27] tested ten cumyl derivatives (Cumyl-BICA, Cumyl-PICA, 5F-Cumyl-PICA, Cumyl-FUBICA, Cumyl-CHMICA, Cumyl-BINACA, Cumyl-PINACA, 5F-Cumyl-PINACA, Cumyl-FUBINACA and Cumyl-CHMINACA) in vitro by using mouse AtT-20 neuroblastoma cells, and all cumyl derivatives activated CB_1_ and CB_2_ receptors with greater potency than CP-55,940, except Cumyl-FUBICA (membrane potential was measured using an FLIPR membrane potential assay kit). The 5F-Cumyl-PINACA was the most potent synthetic cannabinoid as compared to the other cumyl derivatives tested by Longworth et al. [27]. Gamage et al. [28] investigated the receptor binding and agonist stimulated binding of several synthetic cannabinoids, including the indole analogs 5F-Cumyl-PICA and Cumyl-PICA. All tested compounds showed agonistic mode of action at both cannabinoid receptors, but surprisingly the two cumyl derivatives were less potent than THC and most of the other synthetic cannabinoids at CB_1_ and CB_2_ receptors using the [^35^S]GTP_γ_S binding assay. In addition, the cumyl derivatives were less potent than the tested valine derivatives (MMB-FUBINACA and MDMB-FUBINACA) at CB_1_ when using a cAMP assay. Asada et al. [29] did also investigate the CB_1_ and CB_2_ receptor activities of several cumyl derivatives (Cumyl-PINACA, 5F-Cumyl-PINACA, Cumyl-PICA, 5F-Cumyl-PICA, Cumyl-THPINACA, Cumyl-BICA and 5F-Cumyl-P7AICA) using the [^35^S]GTP_γ_S binding assay. All tested compounds were agonists at both cannabinoid receptors, but again some cumyl derivatives (Cumyl-PINACA, 5F-Cumyl-PINACA, Cumyl-PICA and 5F-Cumyl-PICA) showed less activities as compared to CP-55,940. The differing results of these studies are most probably due to differences in receptor expression between different cell lines and different signaling pathways used in the assays.

### Serum concentration course after controlled self-administration of Cumyl-PINACA

**Fig. 2:**
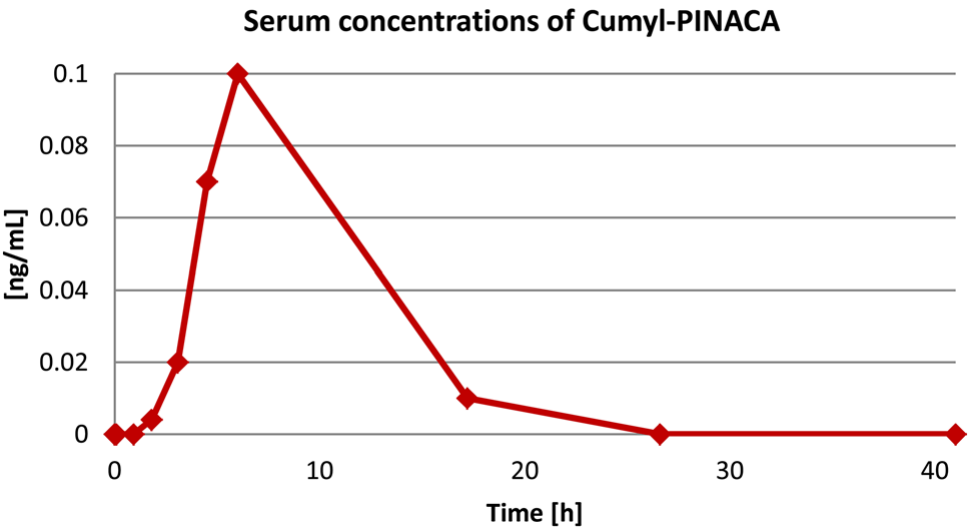
Serum concentrations of Cumyl-PINACA after oral ingestion of 0.6 mg of the compound in a volunteer

After oral ingestion of 0.6 mg Cumyl-PINACA, the volunteer did not experience any drug-related symptoms as expected and intended. Cumyl-PINACA could be detected in serum over a period of about 17 h. The maximum concentration measured was 0.1 ng/mL (6 h after ingestion, Fig. 2).

### Tentative phase I metabolism of Cumyl-PINACA and 5F-Cumyl-PINACA in vivo

**Fig. 3:**
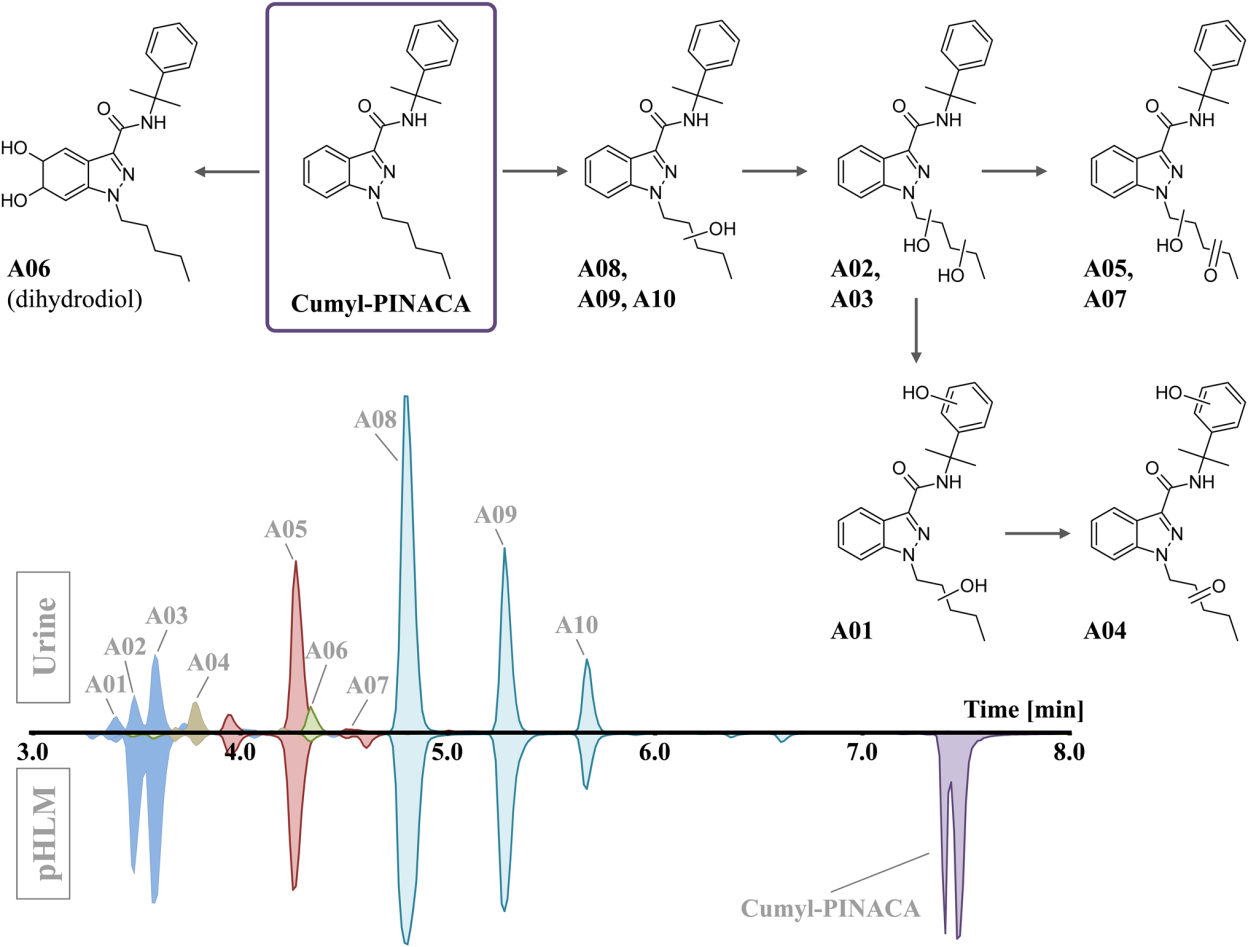
Postulated phase I main metabolites of Cumyl-PINACA as detected in urine after oral ingestion in a volunteer. The corresponding signals obtained by analysis of pooled human liver microsome (pHLM) samples are given for comparison. The position of the dihydrodiol function at the indazole ring for A06 is exemplary and was not confirmed

**Fig. 4:**
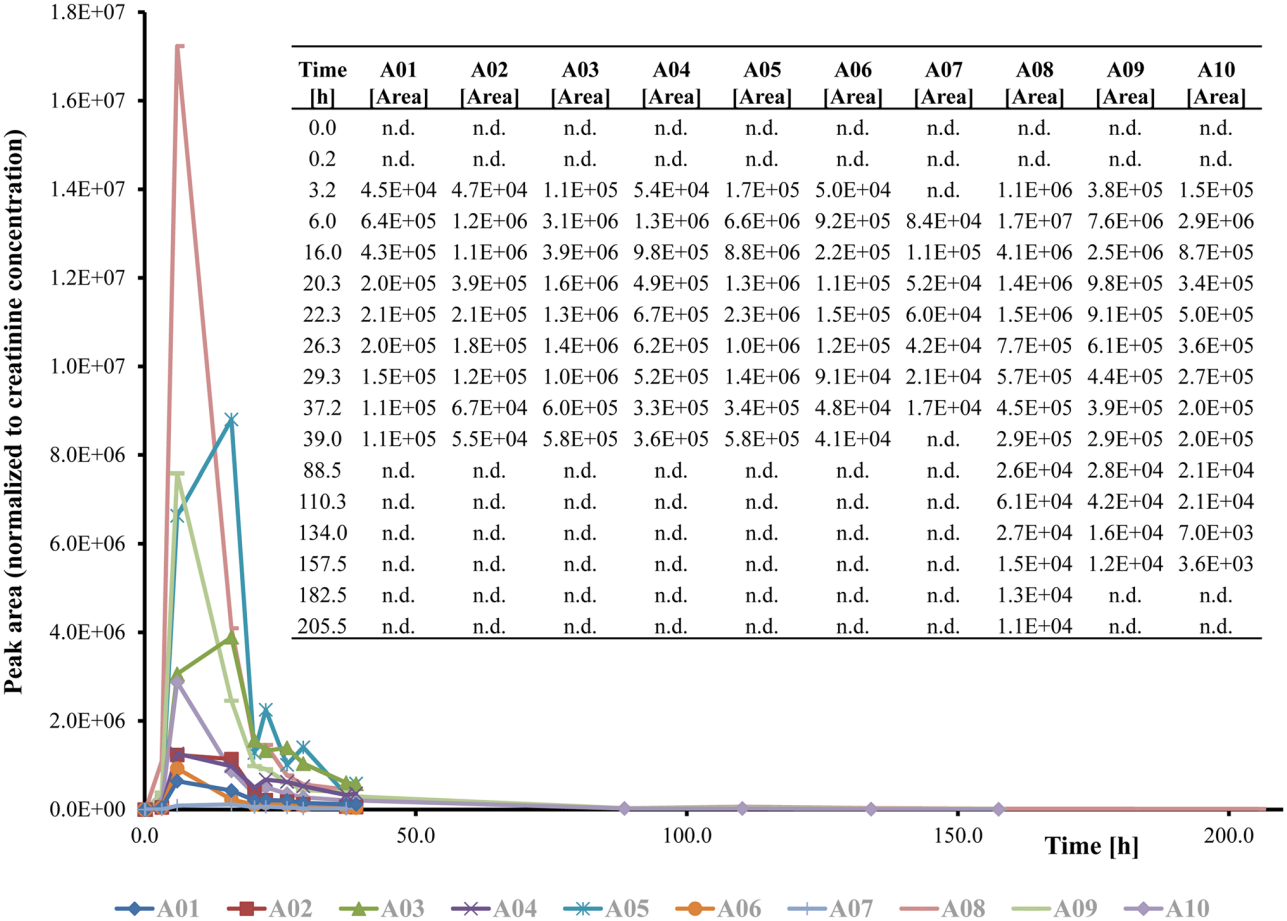
Detection windows of the detected Cumyl-PINACA phase I main metabolites in urine after oral ingestion of 0.6 mg Cumyl-PINACA. The first urine sample was taken directly before the intake and served as a negative control. The second urine sample was taken shortly after the ingestion. *n.d.* not detected

Ten phase I metabolites of Cumyl-PINACA were tentatively identified in urine samples obtained after oral ingestion (Fig. 3). The observed metabolic pathways were dominated by CYP-mediated single and multiple oxidation of the *N*-pentyl side chain. Hydroxylation of the cumyl moiety and formation of a dihydrodiol at the indole ring were also detected with relatively high abundances. The parent compound was not detected in urine. All detected metabolites could be verified in vitro by corresponding signals in the incubated pHLM samples (matching retention times, ion ratios and product ion scans). Comparing the in vitro metabolism of several cumyl derivatives published by Staeheli et al. [21], the main metabolites identified in this in vitro assay showed good agreement. The monohydroxylated metabolite A08 was the most abundant metabolite in all analyzed urine samples. This analyte was detectable in urine for over 8 days after the self-administration and showed the largest detection window of all metabolites (Fig. 4). Therefore, compound A08 is suggested as suitable target for urine analysis when maximum sensitivity is required, e.g., for drug abstinence control testing. Product ion spectra of Cumyl-PINACA and its metabolites are provided in Supplementary Fig. S1. Details on the Cumyl-PINACA metabolic profile detected in urine and pHLM samples are shown in Supplementary Table S3.

**Fig. 5:**
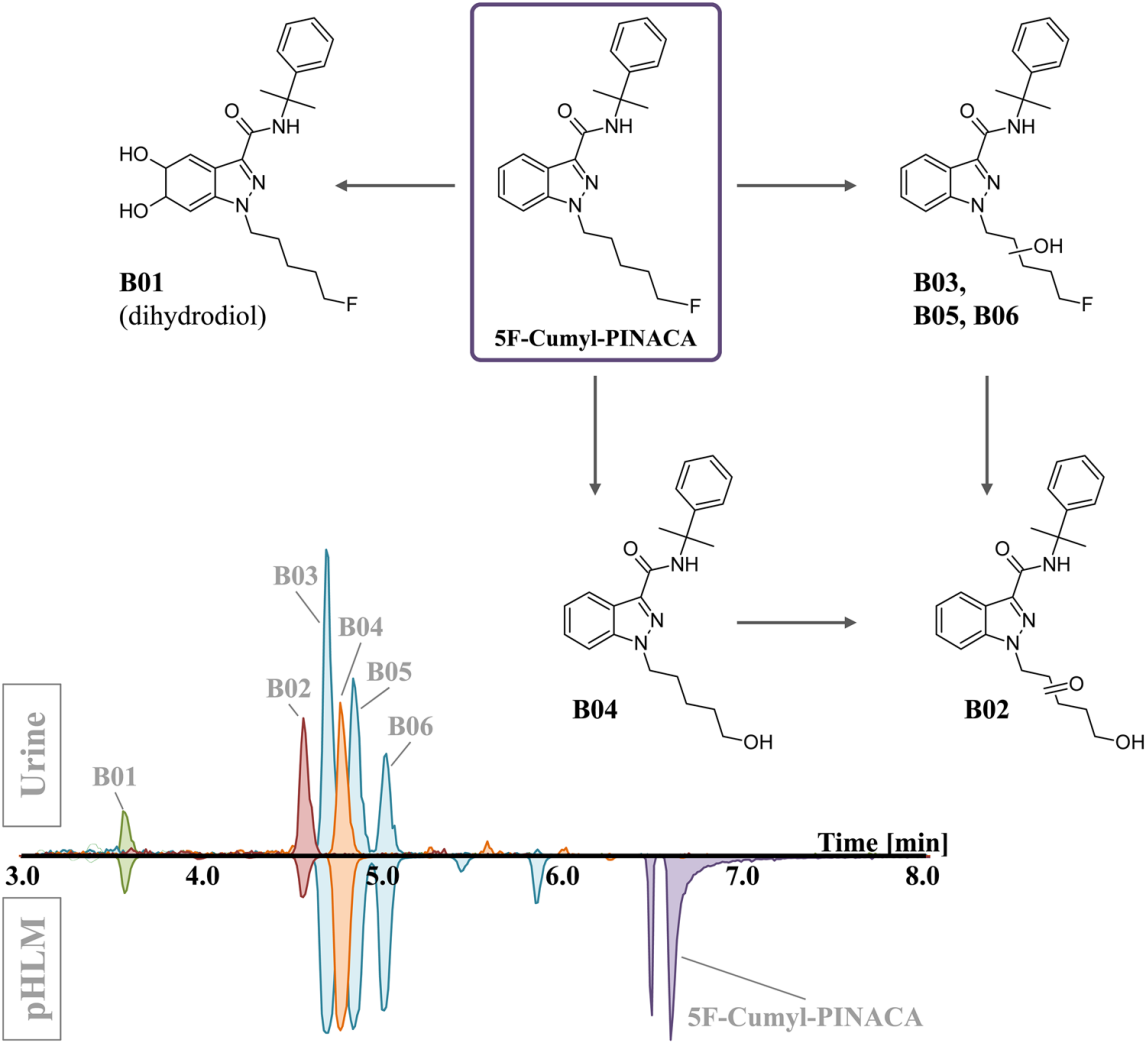
Postulated phase I main metabolites of 5F-Cumyl-PINACA as detected in an authentic urine sample. The corresponding signals obtained by analysis of pHLM samples are given for comparison. The position of the dihydrodiol function at the indazole ring for B01 is exemplary and was not confirmed

Six phase I metabolites of 5F-Cumyl-PINACA were tentatively identified in an authentic urine sample from routine casework (Fig. 5). Similar to its non-fluorinated analog Cumyl-PINACA, three mono-hydroxypentyl isomers (B03, B05, B06) and one dihydrodiol (B01) were detected. The parent compound was not detected. As reported for other synthetic cannabinoids carrying a *N*-(5-fluoropentyl) side chain (e.g., AM-2201 [30], 5F-AB-PINACA [31] and THJ-2201 [32]), the *N*-(5-hydroxypentyl) metabolite (B04) formed by hydrolytic defluorination was detected with high abundance. Further oxidation of the compound B04 yielded in the metabolite B02 (+14 Da) indicating an additional carbonyl function. Following the metabolism data of structurally related substances from the literature, compound B02 is most probably the often appearing *N*-pentanoic acid metabolite [30–33]. All detected 5F-Cumyl-PINACA metabolites were also tentatively identified in vitro by corresponding signals in the pHLM samples. Although further authentic urine samples should be analyzed in order to determine the inter-individual variation in the urinary metabolite profile of 5F-Cumyl-PINACA, the analytes B03, B04 and B05 seem to be suitable markers for a reliable and sensitive detection in urine. Product ion spectra of 5F-Cumyl-PINACA and its metabolites are provided in Supplementary Fig. S4. Details on the 5F-Cumyl-PINACA metabolic profile detected in the authentic urine specimen as well as a pHLM sample are shown in Supplementary Table S5.

It has to be noticed that hydrolytic defluorination of 5F-Cumyl-PINACA (B04) and further oxidation (B02) led to metabolites also detected after ingestion of Cumyl-PINACA (A07 and A08) with corresponding retention time, ion ratios and product ion spectra. The *N*-(5-hydroxypentyl) (A08/B04) as well as the postulated *N*-pentanoic acid (A07/B02) metabolite is a common biotransformation product of both synthetic cannabinoids and therefore—if detected alone—does not allow for clear differentiation between a consumption of 5F-Cumyl-PINACA and the non-fluorinated analog Cumyl-PINACA. Specific metabolites of each compound, such as A09 for Cumyl-PINACA or B03 for 5F-Cumyl-PINACA, should be included in screening methods to facilitate differentiation.

There are some limitations of the study approach that have to be mentioned; the relative abundance of the detected metabolites may be strongly biased by the extraction procedure and matrix effects. Urine samples from only one individual were available to study the metabolic profiles in vivo. Therefore, inter-individual variation of drug metabolism (e.g., by genetic polymorphisms, etc.) cannot be discussed. Analysis of further positive samples from several individuals should be performed in order to assess the robustness of the suggested target analytes. Elucidation of the exact chemical structure of the postulated metabolites would require synthesis of reference material and remains to be investigated. It can be expected that phase I metabolites of 5F-/Cumyl-PINACA will be extensively conjugated (e.g., glucuronidation) prior to urinary elimination, as described for other synthetic cannabinoids [33–35]. Hence, cleavage of conjugates should be performed during sample preparation when the discussed metabolites are used as target for drug detection in urine.

## Conclusions

For the first time, the new synthetic cannabinoid 5F-Cumyl-PINACA was identified in e-liquids as a rather new application form. Besides the two substances described here, nine further structures carrying a cumyl moiety were reported to the EMCDDA so far.

The presented results of the cAMP biosensor assay suggest that the cumyl moiety (Cumyl-PINACA)—when compared to a naphthoyl moiety (JWH-018) or a valine moiety—leads to a significantly higher CB_1_ receptor-mediated potency. It remains an open question to be targeted by further studies if the cumyl moiety does in general lead to relatively potent synthetic cannabinoid receptor agonists.

The results of the in vitro incubation of both cumyl derivatives with pHLM were in good agreement with the metabolic profiles observed in vivo and demonstrated that pHLM are a powerful tool for the investigation of synthetic cannabinoid metabolism. The tentative identification of the in vivo metabolites proved to be very reliable despite the fact that a low-resolution LC–MS/MS instrument was used in this study. As described for the biotransformation of structurally related synthetic cannabinoids with a *N*-(5-fluoropentyl) side chain, hydrolytic defluorination and further oxidation led to common metabolites with the non-fluorinated analog which has to be considered for interpretation of metabolite findings in urine samples.

The increasing popularity of e-liquids particularly among young people and the high potency of the synthetic cannabinoids added pose a serious threat to the health of consumers. There is a high risk of unintended poisoning, and in the long term the prevalence of these drugs might rise in the younger population as a consequence of the introduction of trendy products.

To our knowledge, this study is the first trial to present in vivo metabolism of Cumyl-PINACA and 5F-Cumyl-PINACA in humans.

## Electronic supplementary material

Below is the link to the electronic supplementary material.

Supplementary material 1 (PDF 2055 kb)

Supplementary material 2 (PDF 322 kb)
