# Supplementary material for: 5F-Cumyl-PINACA in ‘e-liquids’ for electronic cigarettes: comprehensive characterization of a new type of synthetic cannabinoid in a trendy product including investigations on the in vitro and in vivo phase I metabolism of 5F-Cumyl-PINACA and its non-fluorinated analog Cumyl-PINACA
Source: Forensic Toxicol. 2018 Nov 13;37(1):186–96. doi: 10.1007/s11419-018-0451-8 (PMC6315005; doi:10.1007/s11419-018-0451-8)
Supplement: Supplementary file 1 — Supplementary material 1 (PDF 2055 kb) [file 11419_2018_451_MOESM1_ESM.pdf]

Electronic supplementary material – Forensic Toxicology

5F-Cumyl-PINACA in 'e-liquids' for electronic cigarettes: comprehensive characterization of a new type of synthetic cannabinoid in a trendy product including investigations on the in vitro and in vivo phase I metabolism of 5F-Cumyl-PINACA and its non-fluorinated analog Cumyl-PINACA

Verena Angerer[1,2,†], Florian Franz[1,2,3,†], Bjoern Moosmann[4], Philippe Bisel[5], Volker Auwärter[1,2,]*

[1]Institute of Forensic Medicine, Forensic Toxicology, Medical Center – University of Freiburg, Albertstr. 9, 79104 Freiburg, Germany

[2] Faculty of Medicine, University of Freiburg, Breisacher Str. 153, 79110 Freiburg, Germany

[3]Hermann Staudinger Graduate School, University of Freiburg, Hebelstr. 27, 79104 Freiburg, Germany

[4]Institute of Forensic Medicine, Forensic Toxicology, Kantonsspital St.Gallen, Rorschacher Str. 95, 9007 St.Gallen, Switzerland

[5]Institute for Pharmaceutical Sciences, University of Freiburg, Albertstr. 25, 79104 Freiburg, Germany

* Corresponding author:

V. Auwärter

tel.: +49 761 203 6862

fax: +49 761 203 6826

e-mail: volker.auwaerter@uniklinik-freiburg.de

[†]V. Angerer and F. Franz contributed equally to this work.

Fig. S1 Enhanced Product Ion (EPI) spectra of Cumyl-PINACA metabolites detected in pooled human liver microsome (pHLM) assay samples and authentic urine samples (recorded from urine sample 6 after treatment with glucuronidase) in comparison to the EPI spectrum of the parent compound ‘Cumyl-PINACA’. Proposed structural formulae and fragmentation is shown for each compound along with their molecular formulae, monoisotopic and single-protonated masses. The EPI scans were recorded with a declustering potential (DP) of 40 V, an entrance potential (EP) of 8 V and a collision energy (CE) of 35 V with a collision energy spread (CES) of ±15 V. The position of the dihydrodiol function at the indazole ring for A06 is exemplary and was not confirmed

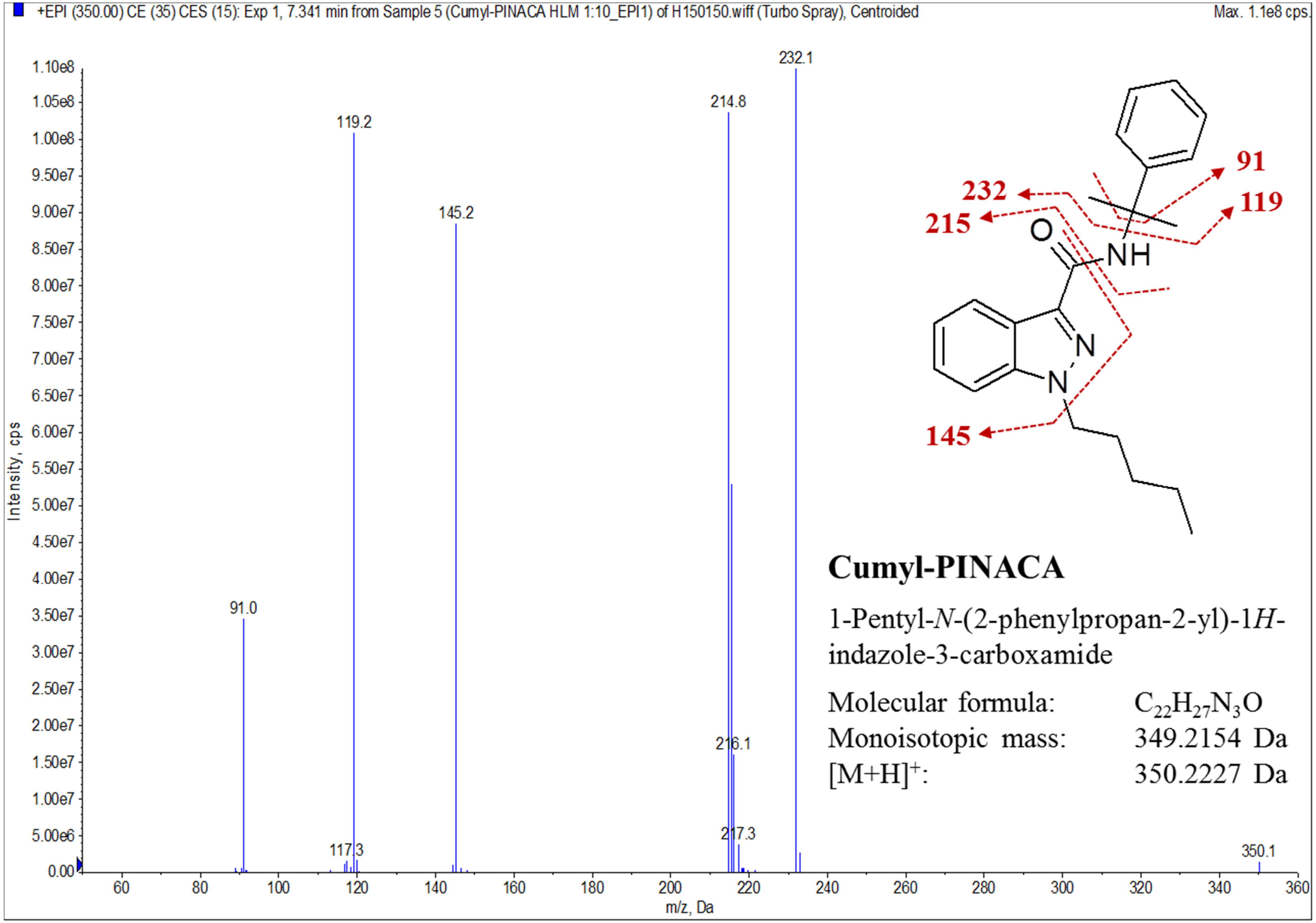

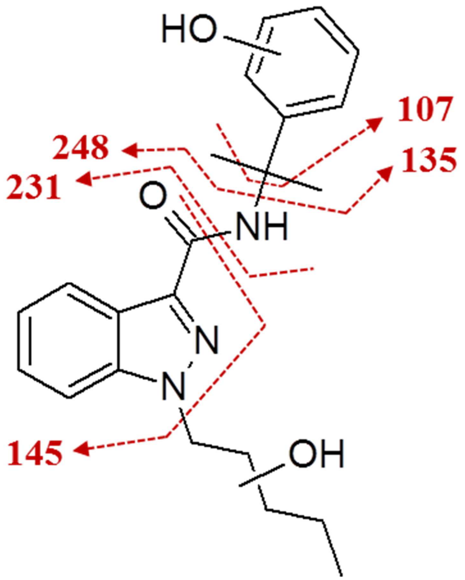

A01 (RT 3.3 min)

Molecular formula:	$C_{22}H_{27}N_3O_3$
Monoisotopic mass:	381.2052 Da
$[M+H]^+$:	382.2125 Da

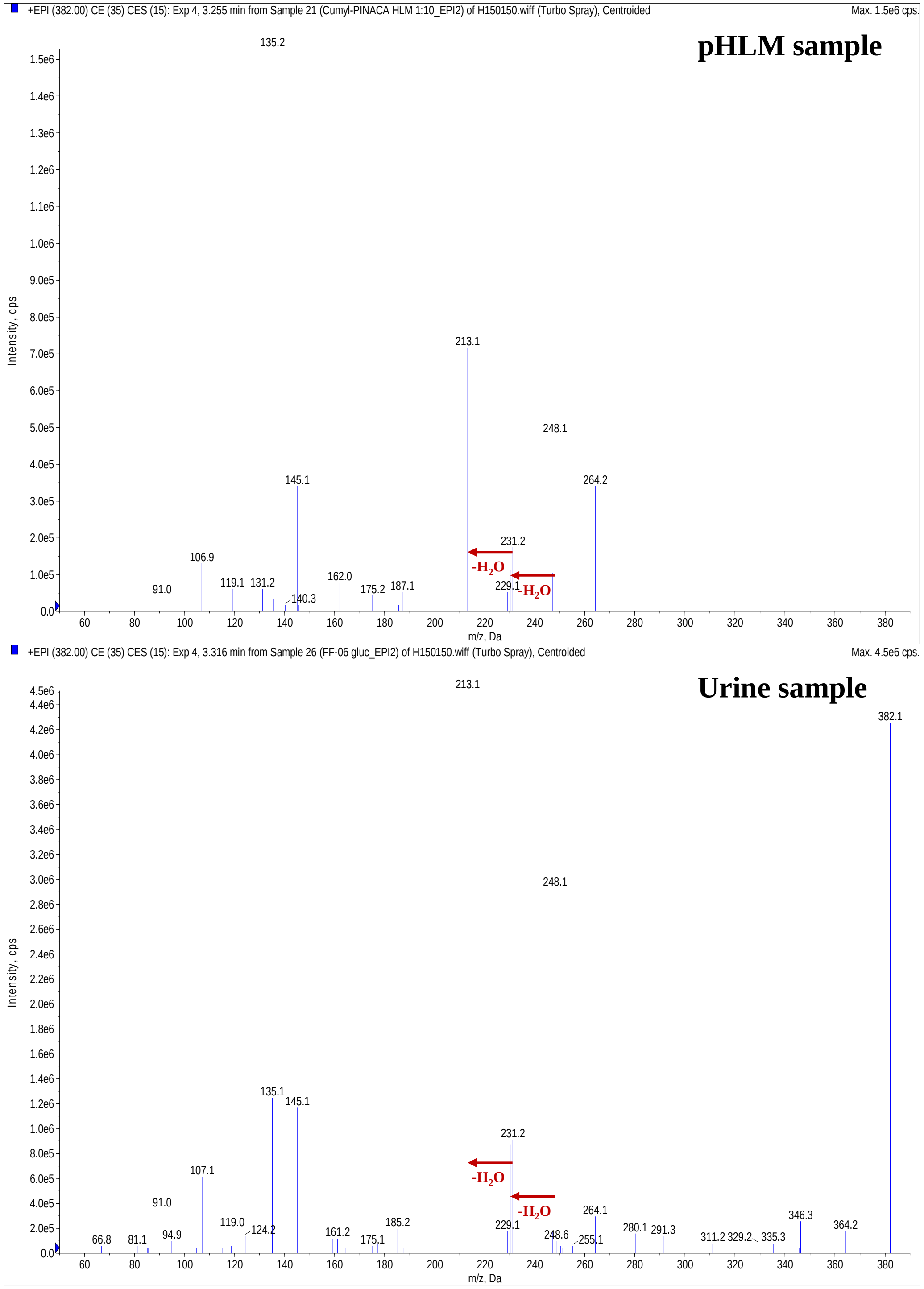

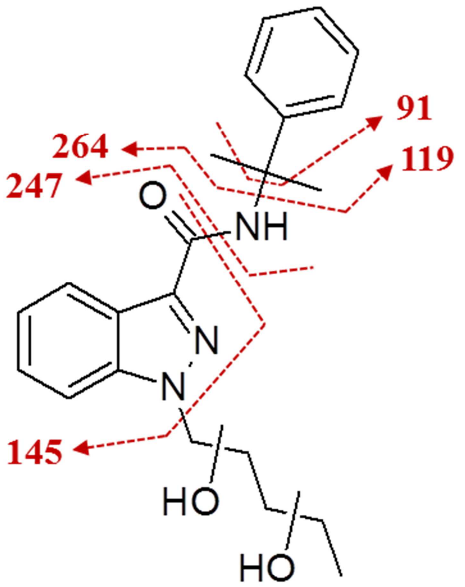

A02 (RT 3.4 min)

Molecular formula:	$C_{22}H_{27}N_3O_3$
Monoisotopic mass:	381.2052 Da
$[M+H]^+$:	382.2125 Da

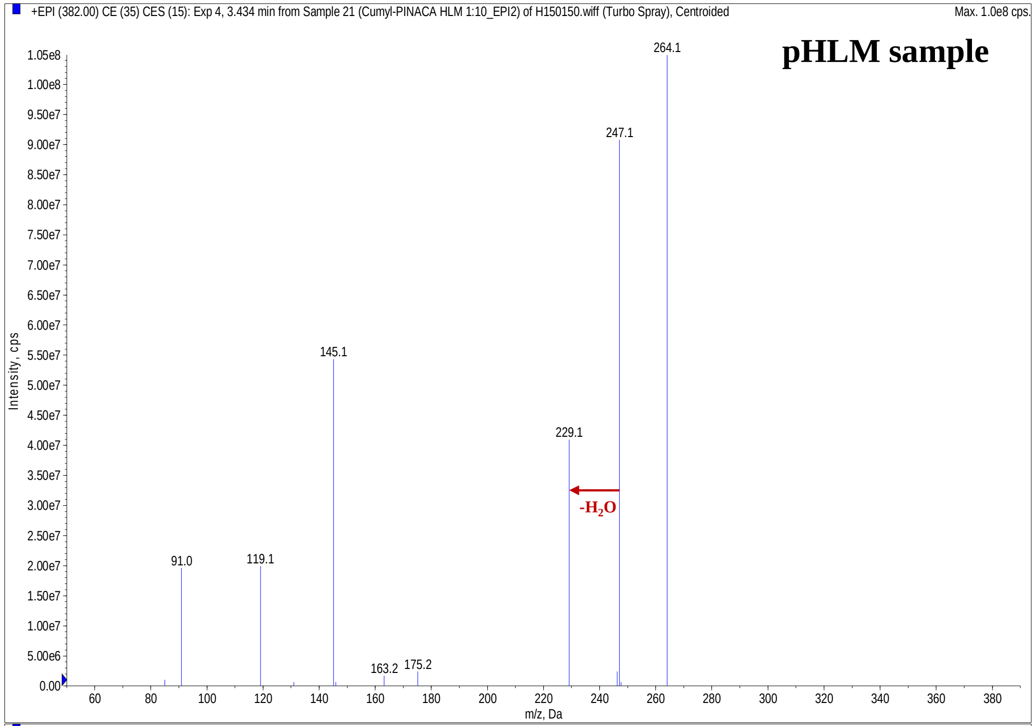

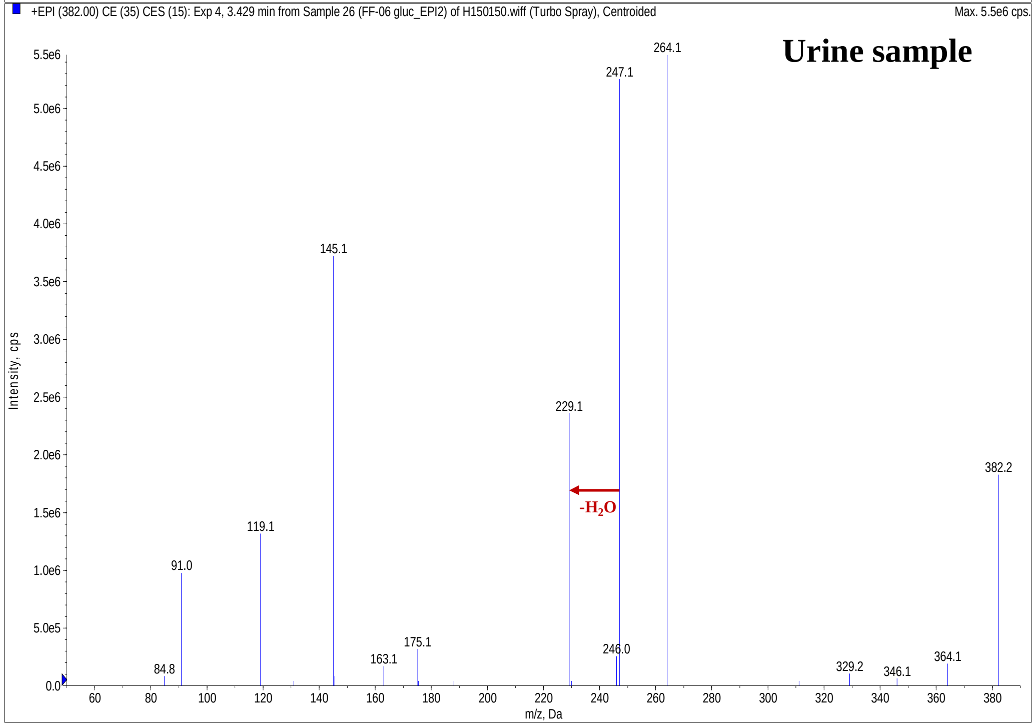

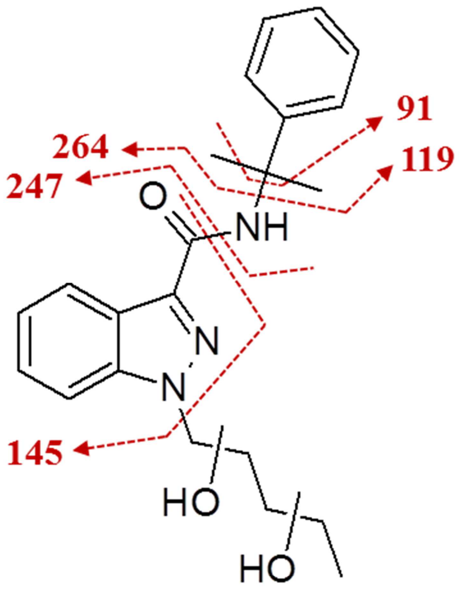

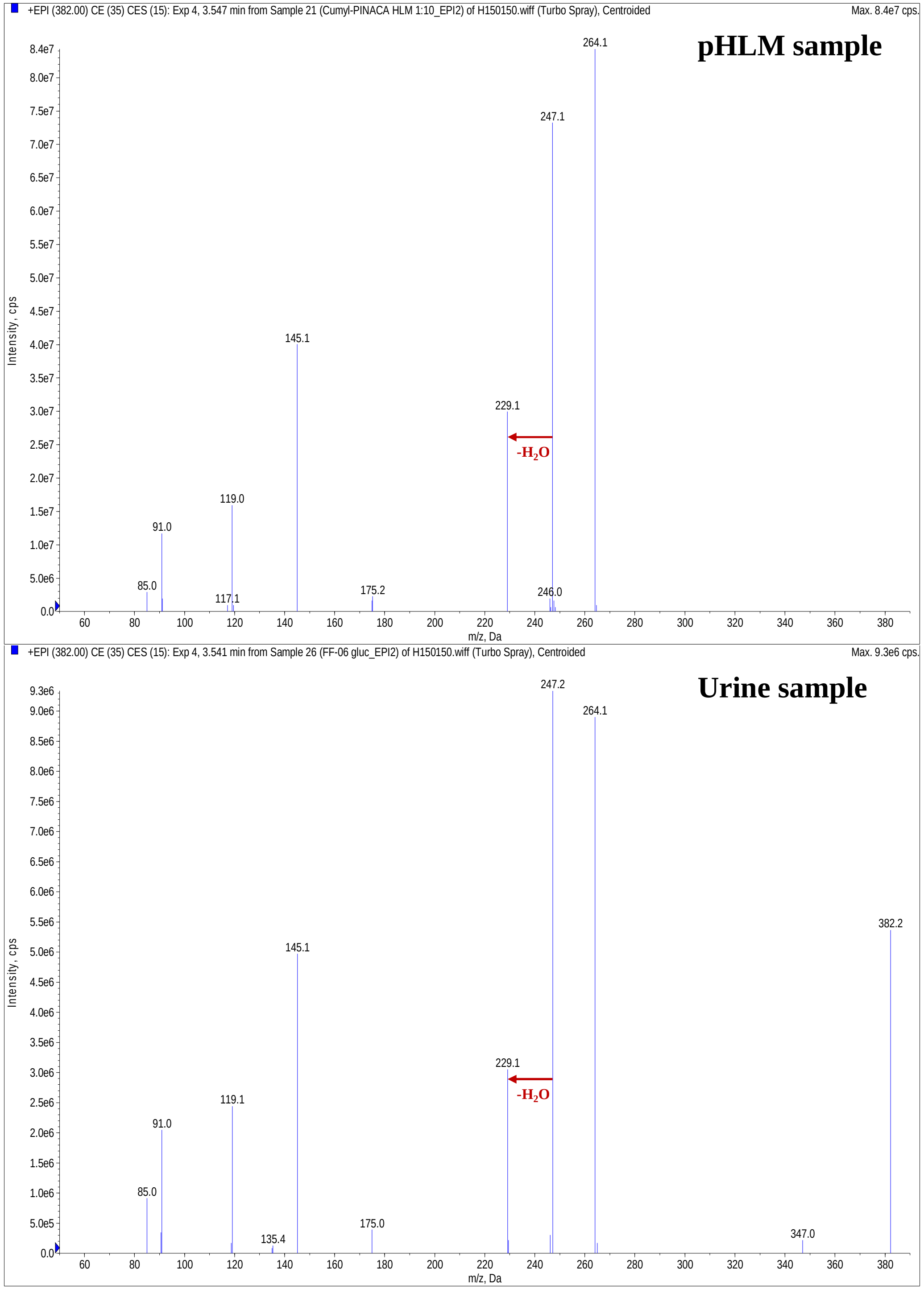

A03 (RT 3.5 min)

Molecular formula:	$C_{22}H_{27}N_3O_3$
Monoisotopic mass:	381.2052 Da
$[M+H]^+$:	382.2125 Da

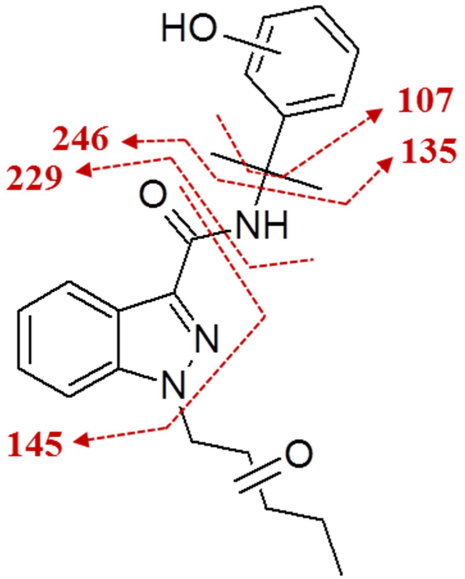

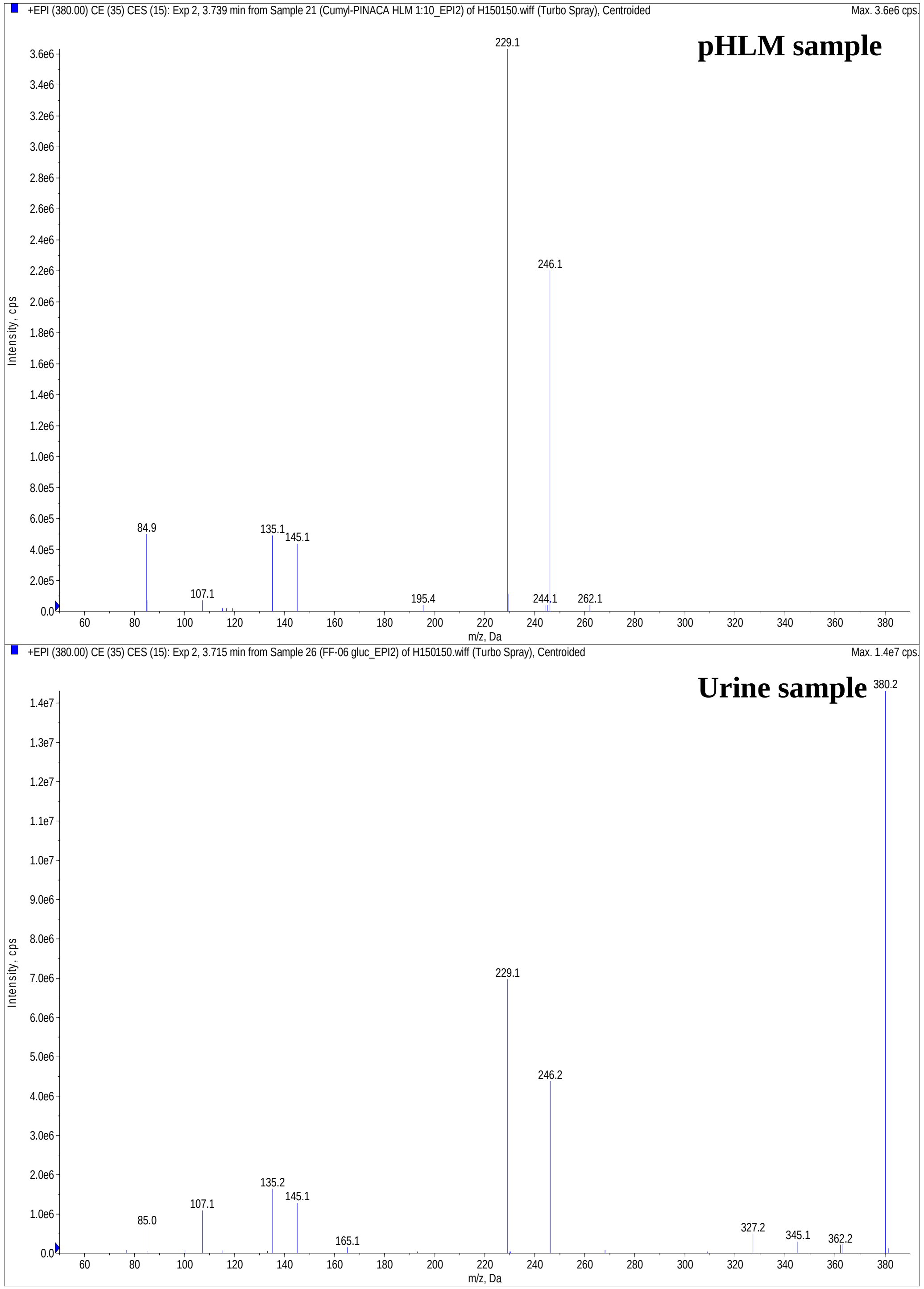

A04 (RT 3.7 min)

Molecular formula:	$C_{22}H_{25}N_3O_3$
Monoisotopic mass:	379.1896 Da
$[M+H]^+$:	380.1969 Da

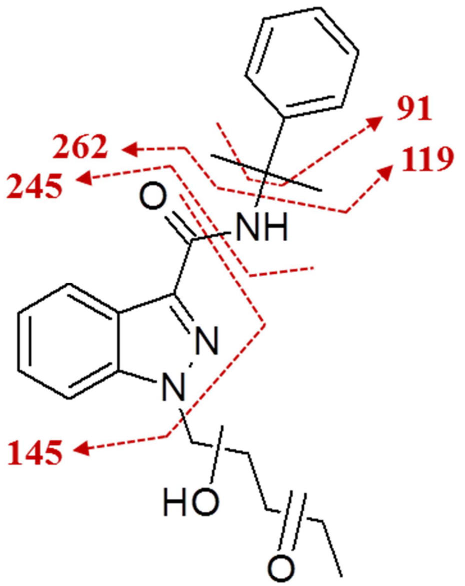

A05 (RT 4.2 min)

Molecular formula:	$C_{22}H_{25}N_3O_3$
Monoisotopic mass:	379.1896 Da
$[M+H]^+$:	380.1969 Da

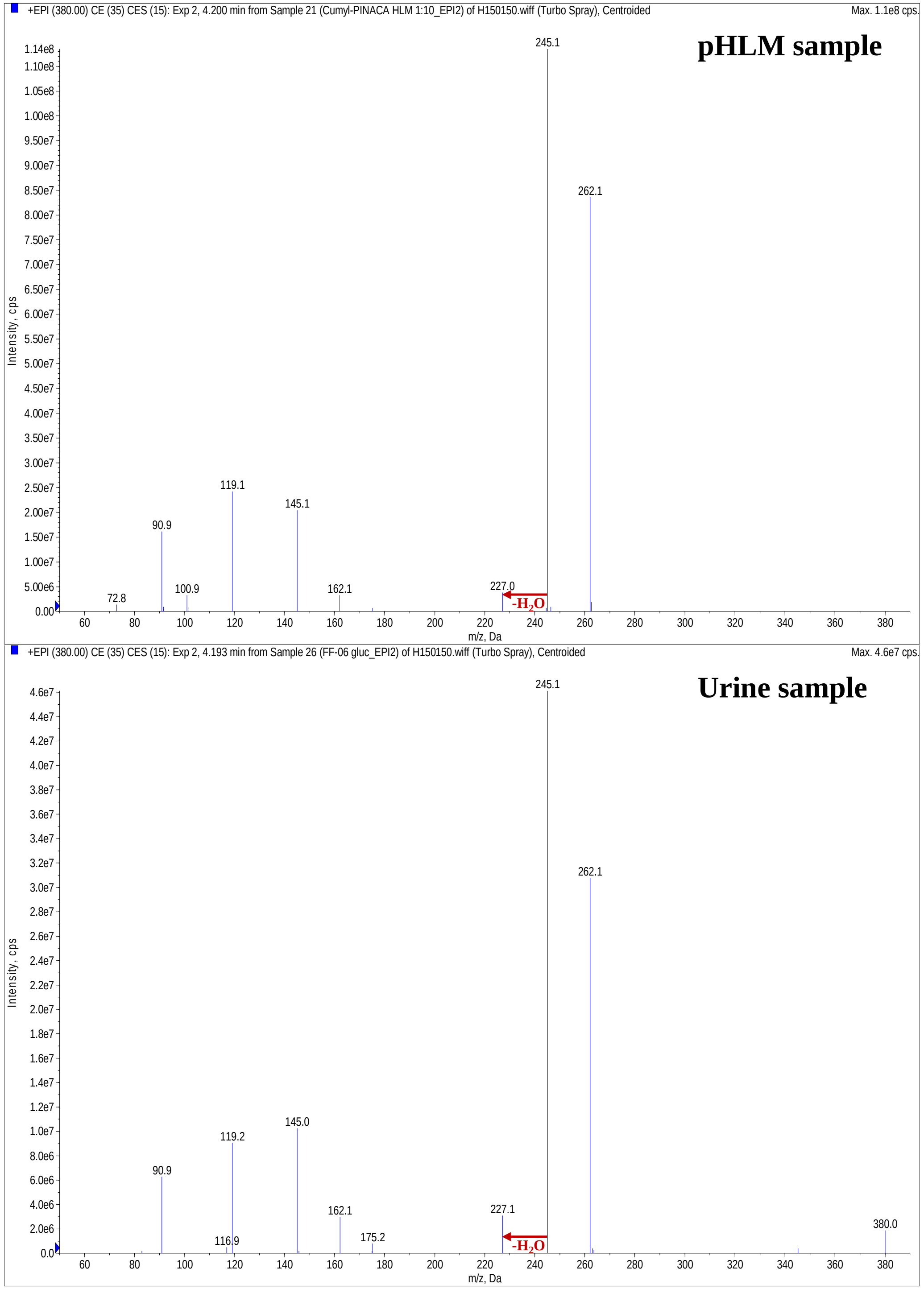

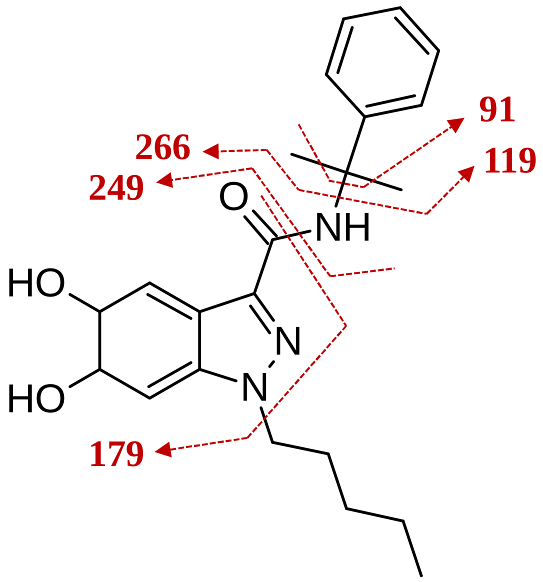

A06 (RT 4.3 min)

(dihydrodiol)

Molecular formula: $C_{22}H_{29}N_3O_3$

Monoisotopic mass: 383.2209 Da

$[M+H]^+$: 384.2282 Da

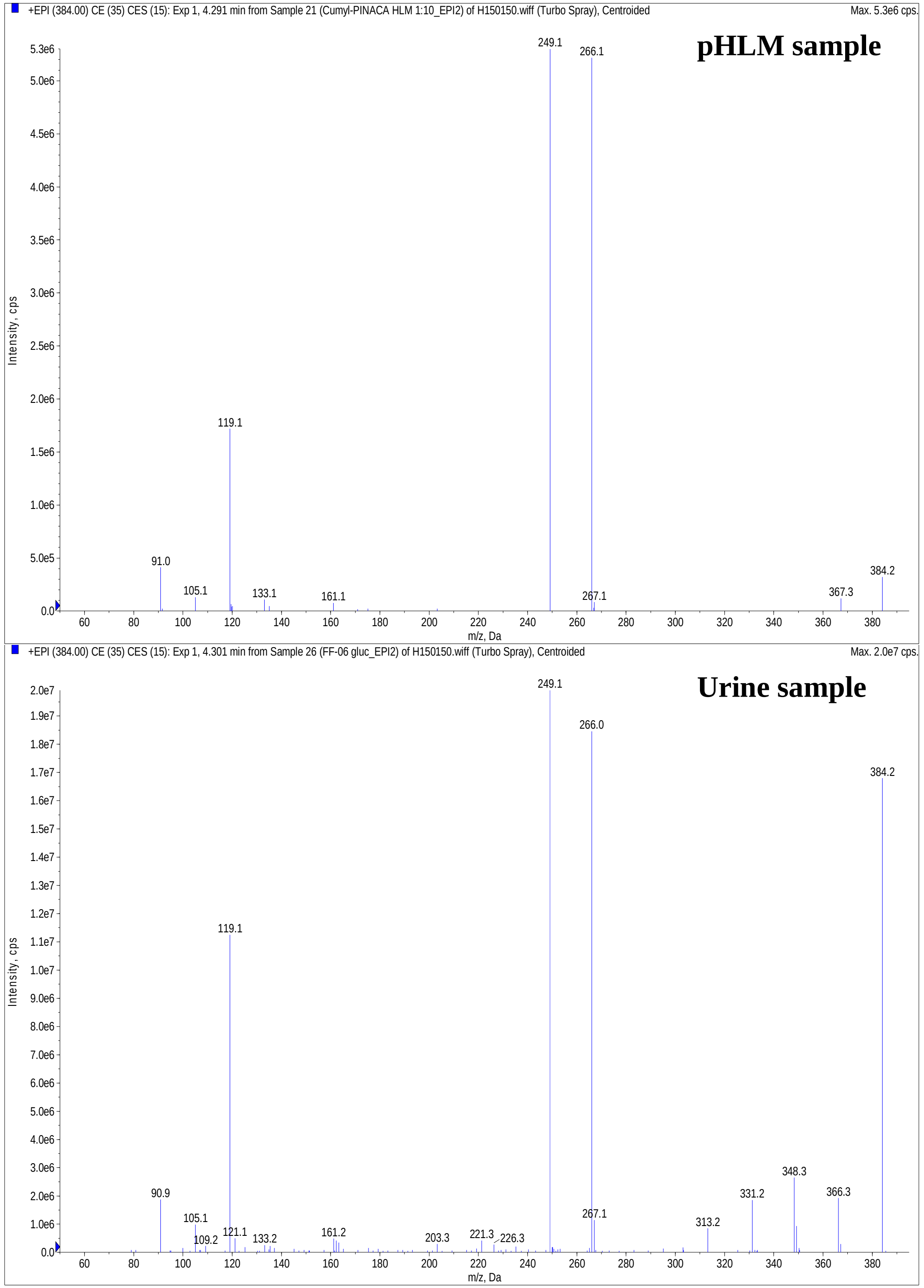

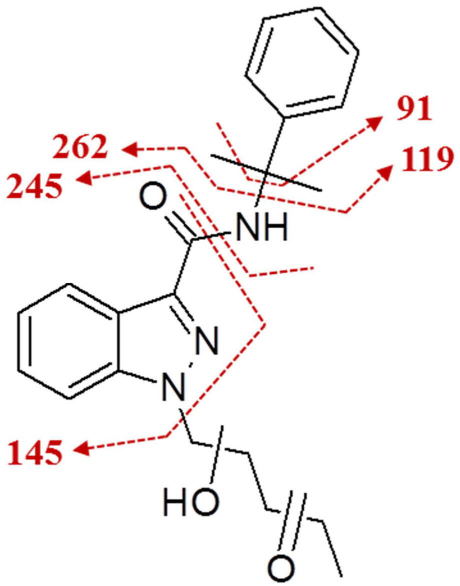

A07 (RT 4.5 min)

Molecular formula:	$C_{22}H_{25}N_3O_3$
Monoisotopic mass:	379.1896 Da
$[M+H]^+$:	380.1969 Da

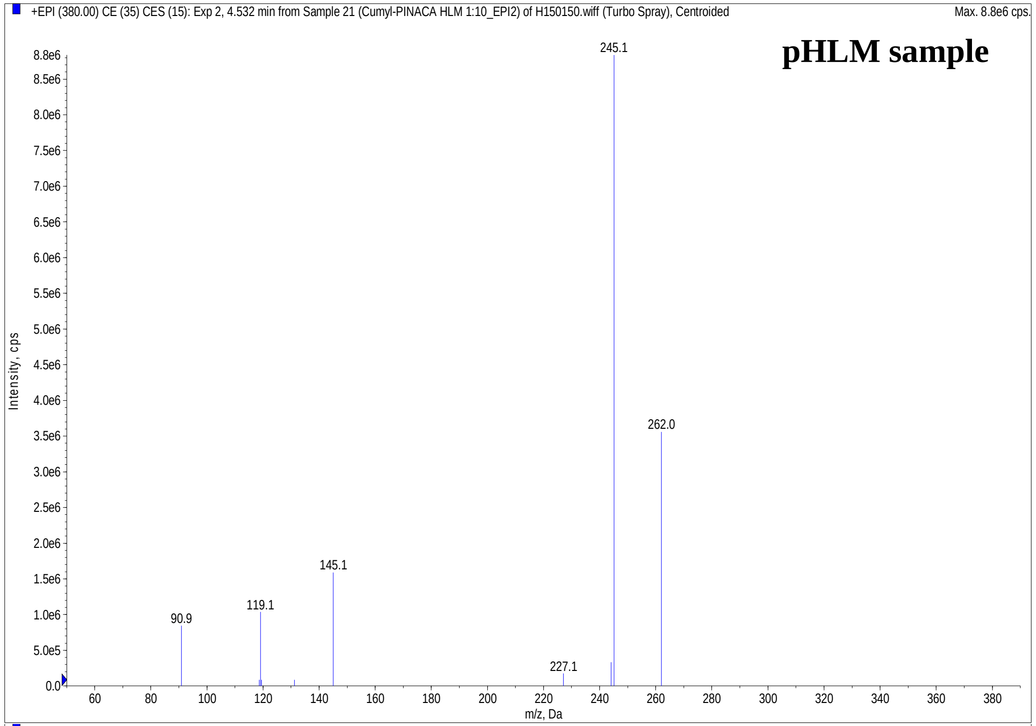

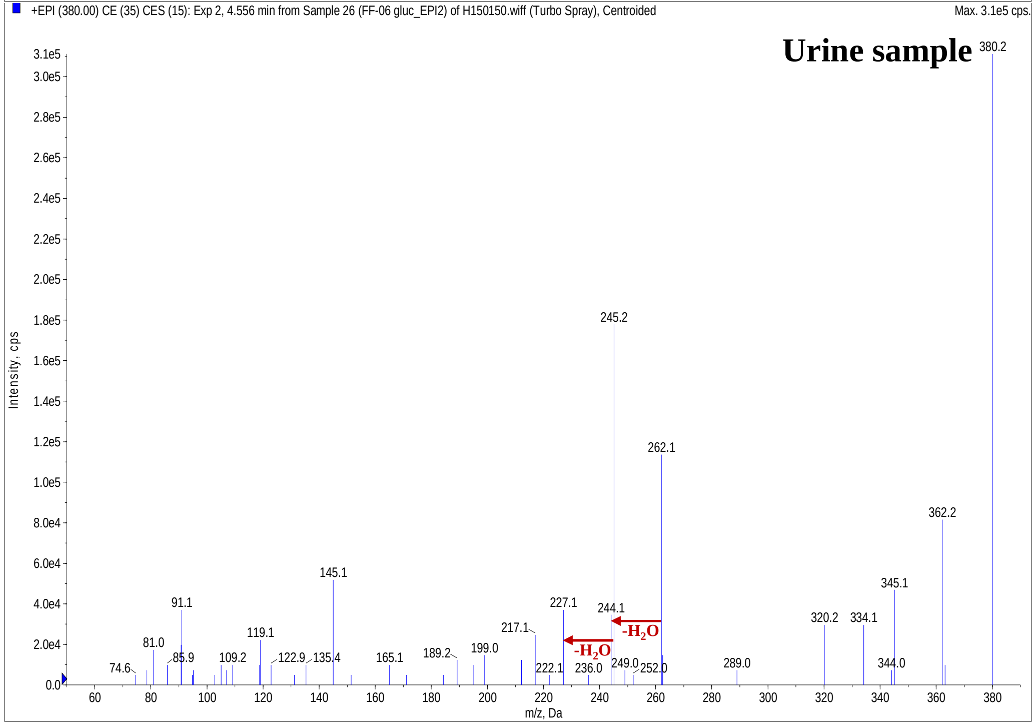

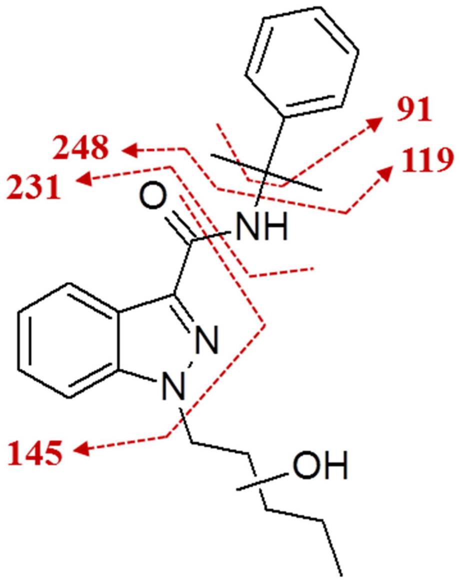

A08 (RT 4.8 min)

Molecular formula:	$C_{22}H_{27}N_3O_2$
Monoisotopic mass:	365.2103 Da
$[M+H]^+$:	366.2176 Da

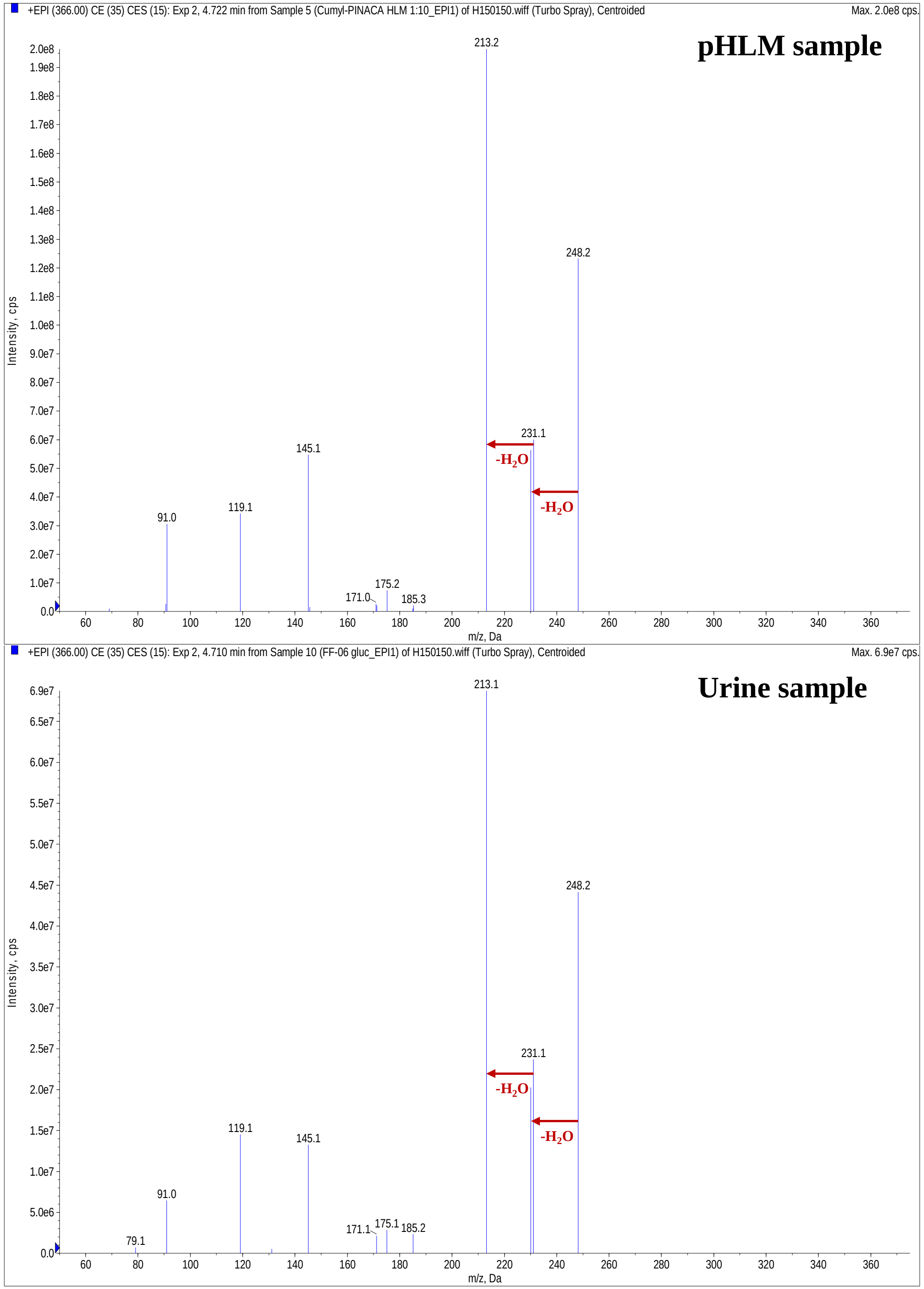

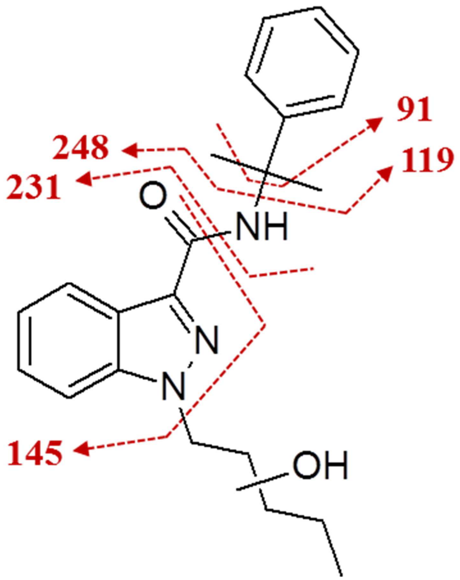

A09 (RT 5.2 min)

Molecular formula:	$C_{22}H_{27}N_3O_2$
Monoisotopic mass:	365.2103 Da
$[M+H]^+$:	366.2176 Da

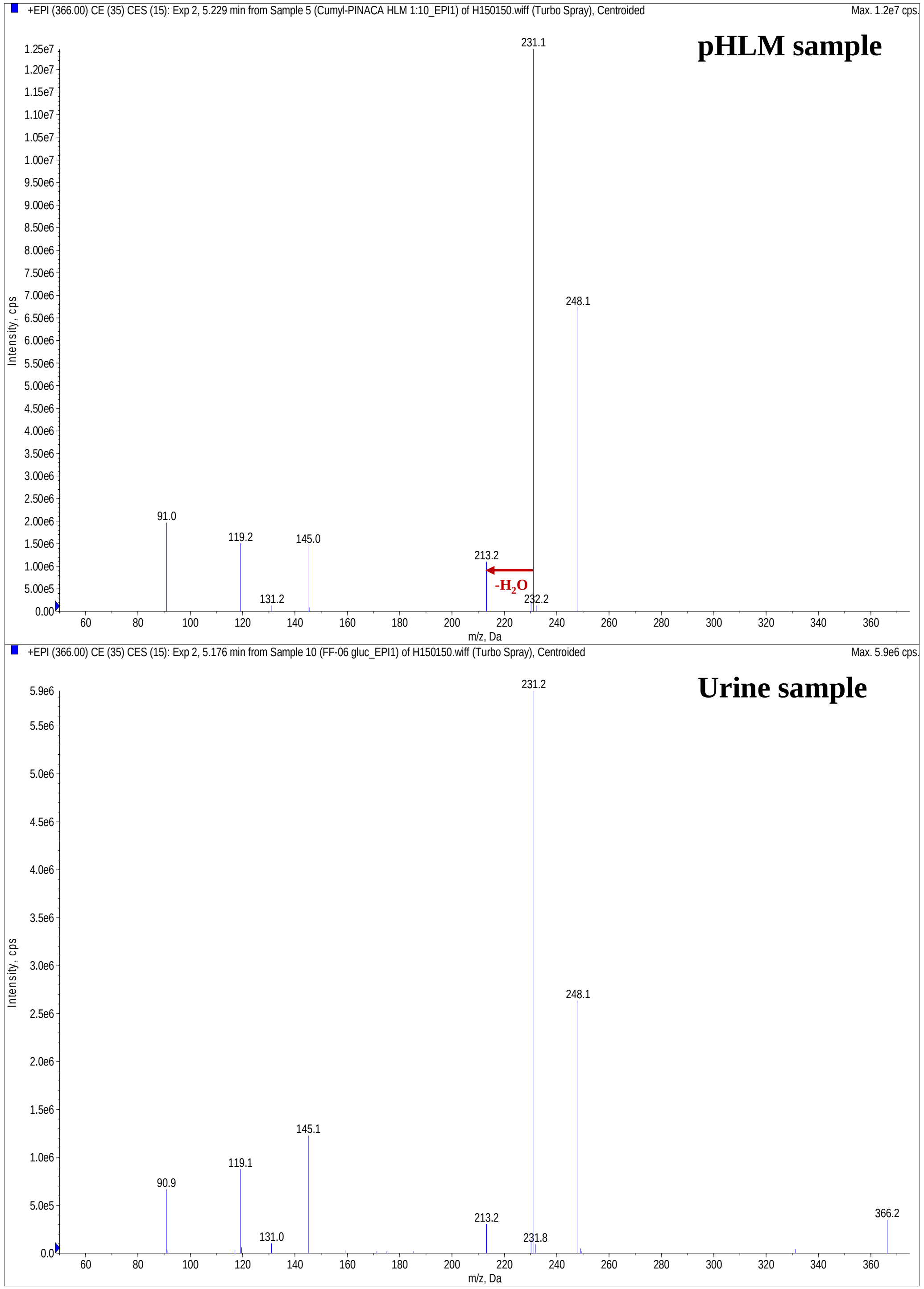

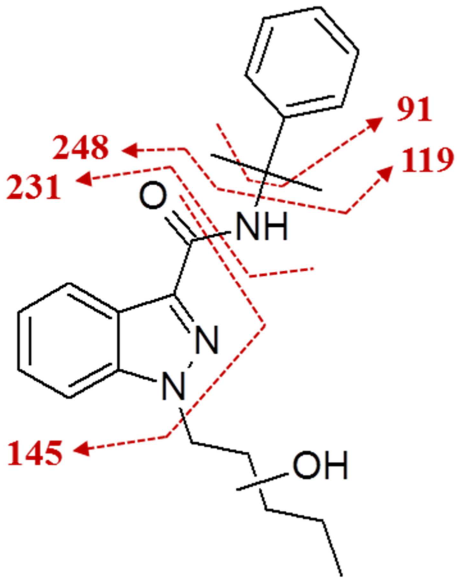

A10 (RT 5.6 min)

Molecular formula:	$C_{22}H_{27}N_3O_2$
Monoisotopic mass:	365.2103 Da
$[M+H]^+$:	366.2176 Da

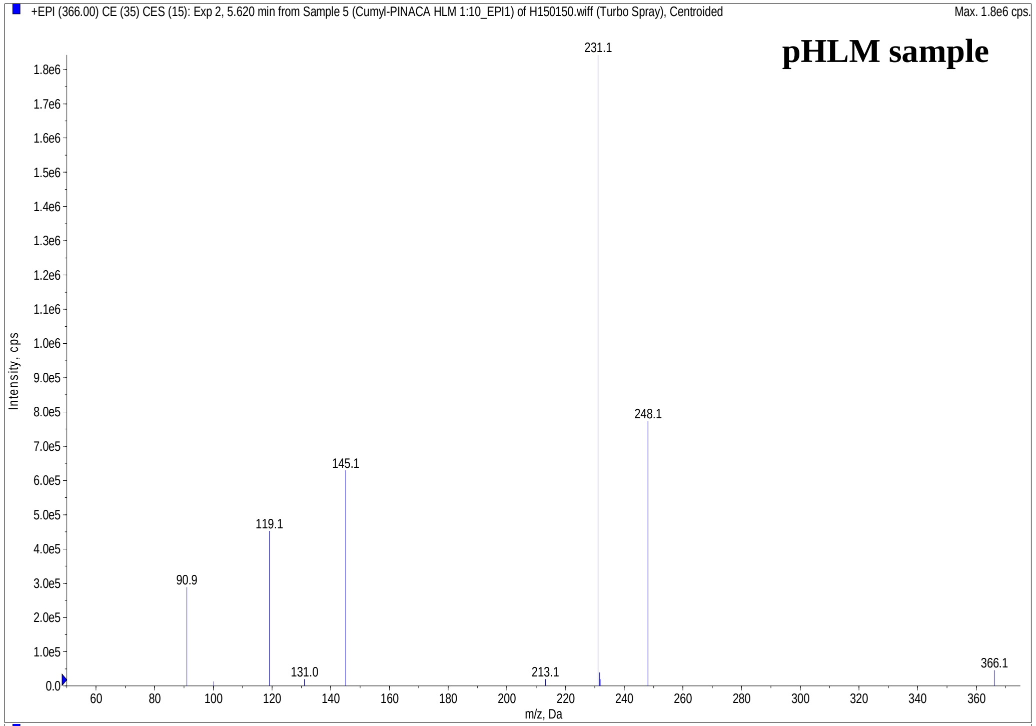

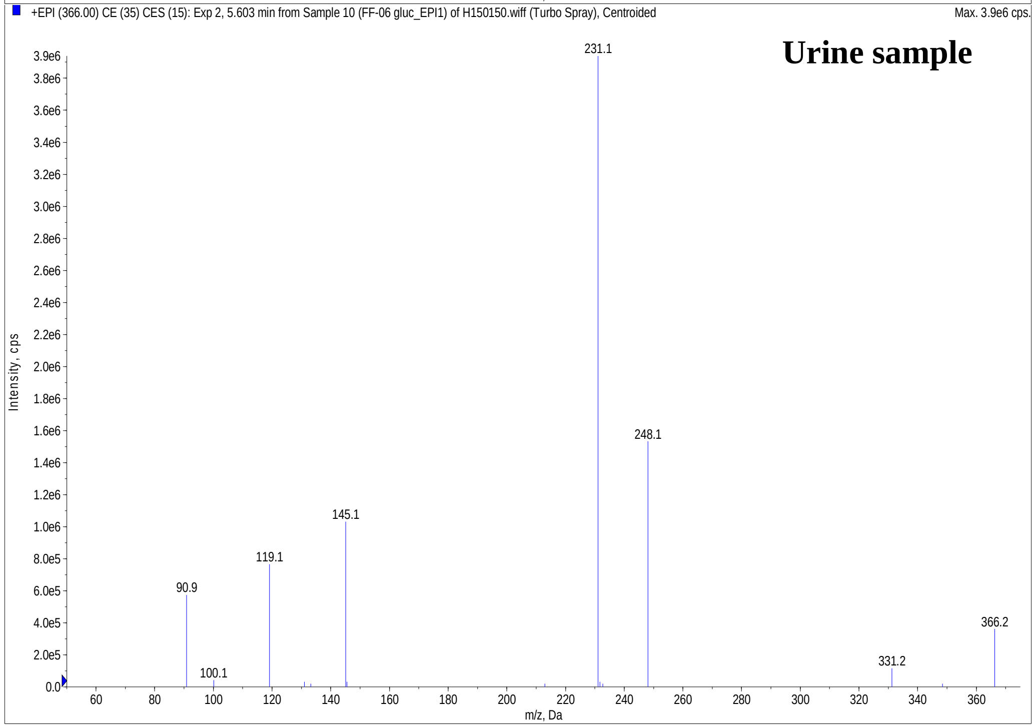

Fig. S2 GC–EI-MS and proposed fragmentation of the unknown compound, later identified as 5F-Cumyl-PINACA (retention time 11.7 min)

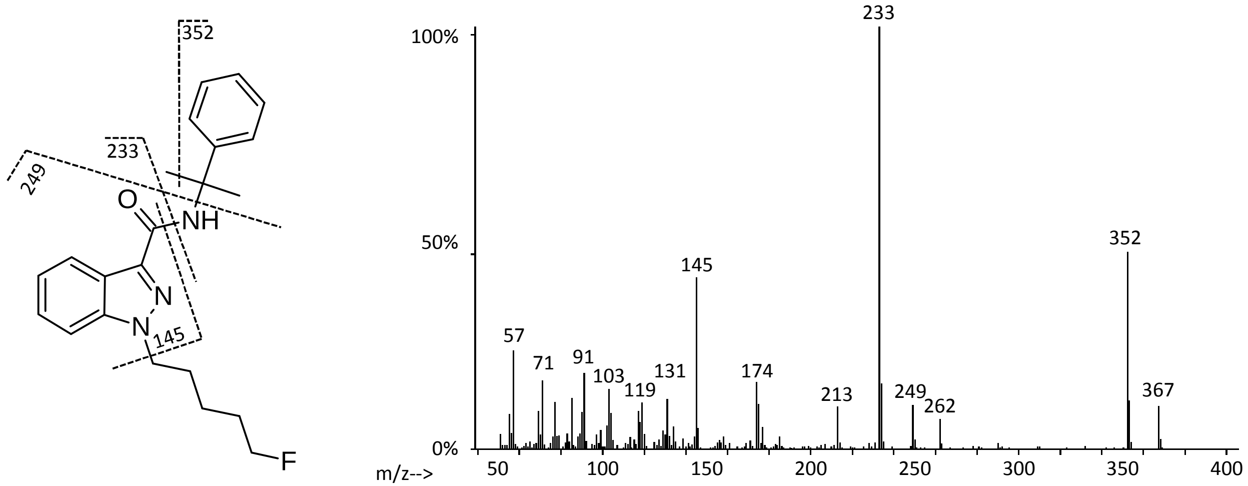

Fig. S3 Structural formula of the unknown compound identified as 5F-Cumyl-PINACA

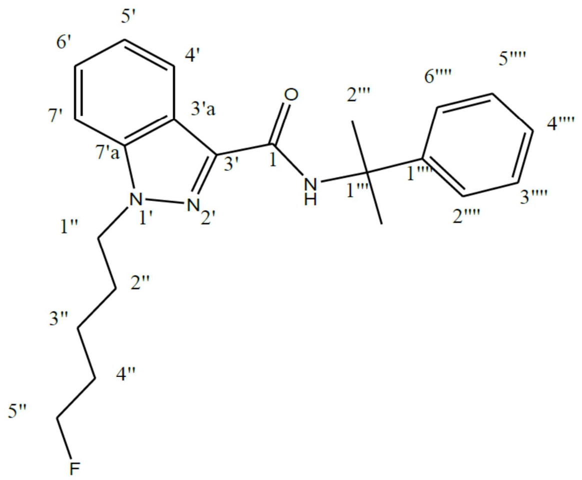

Fig. S4 Enhanced Product Ion (EPI) spectra of 5F-Cumyl-PINACA metabolites detected in pooled human liver microsome (pHLM) assay samples and authentic urine samples (recorded from urine sample 6 after treatment with glucuronidase) in comparison to the EPI spectrum of the parent compound '5F-Cumyl-PINACA'. Proposed structural formulae and fragmentation is shown for each compound along with their molecular formulae, monoisotopic and single-protonated masses. The EPI scans were recorded with a declustering potential (DP) of 65 V, an entrance potential (EP) of 12 V and a collision energy (CE) of 35 V with a collision energy spread (CES) of ±15 V. The position of the dihydrodiol function at the indazole ring for B01 is exemplary and was not confirmed

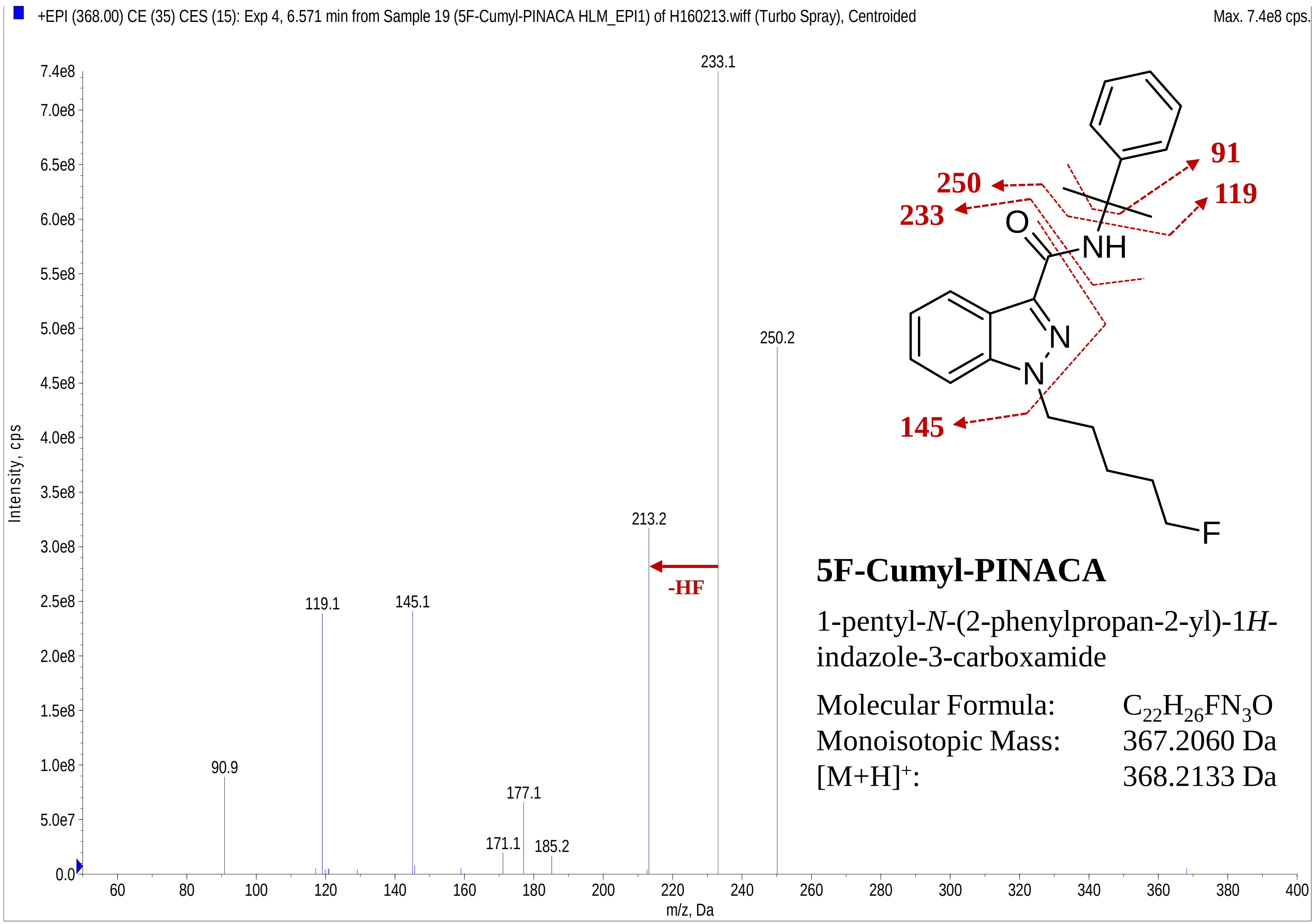

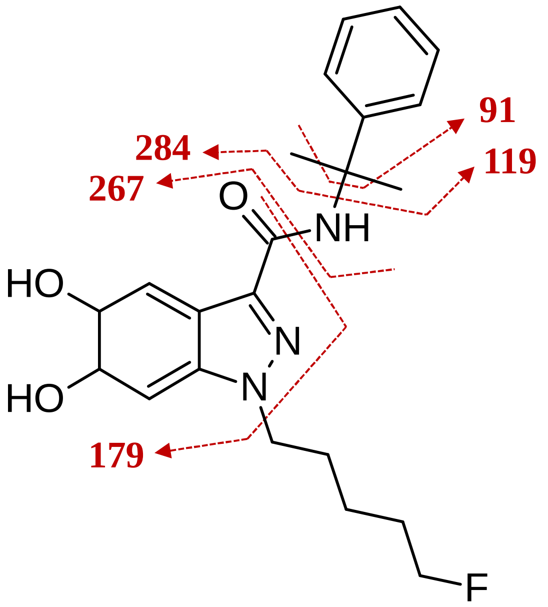

B01 (RT 3.6 min)

(dihydrodiol)

Molecular formula:	$C_{22}H_{28}FN_3O_3$
Monoisotopic mass:	401.2115 Da
$[M+H]^+$:	402.2187 Da

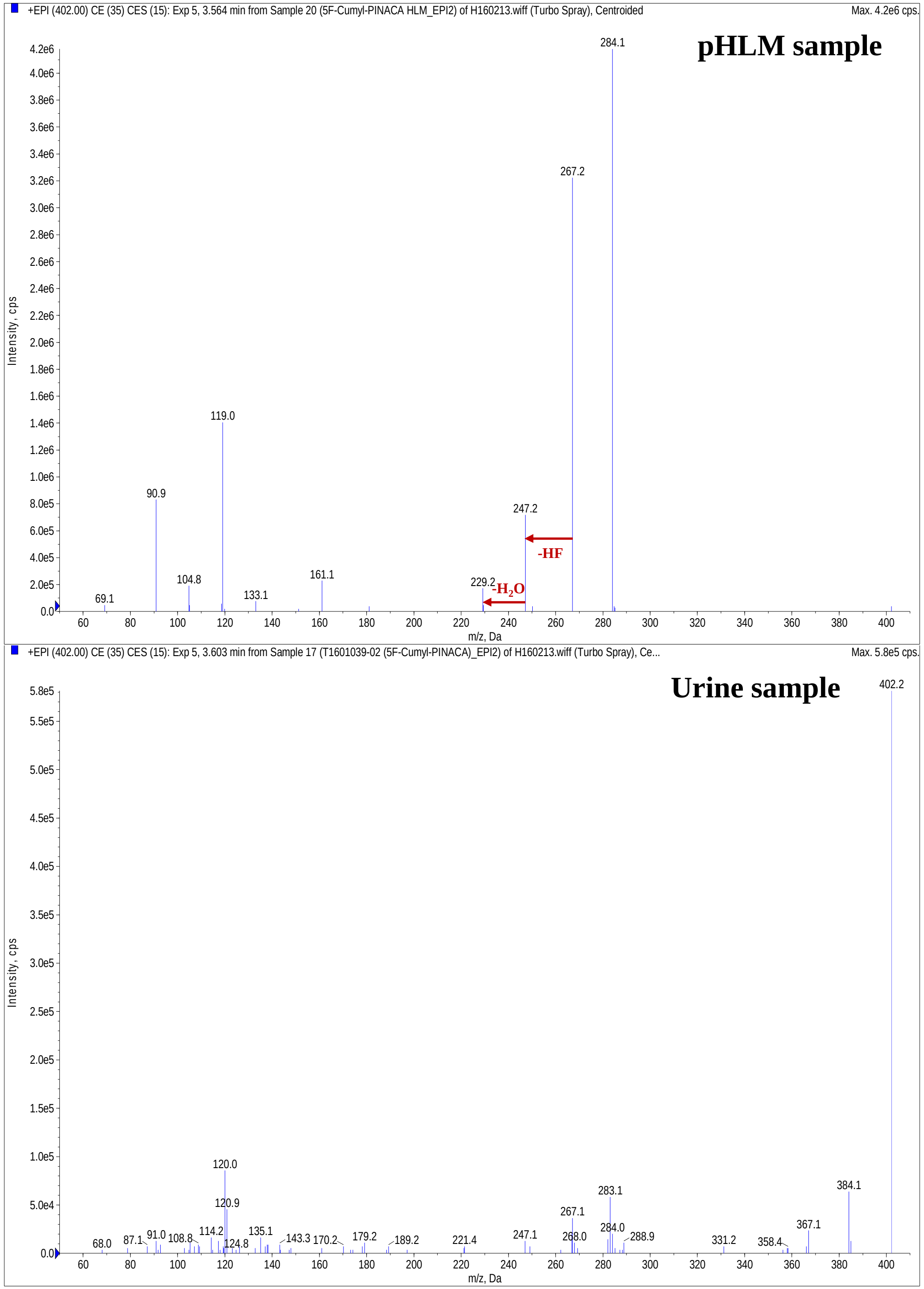

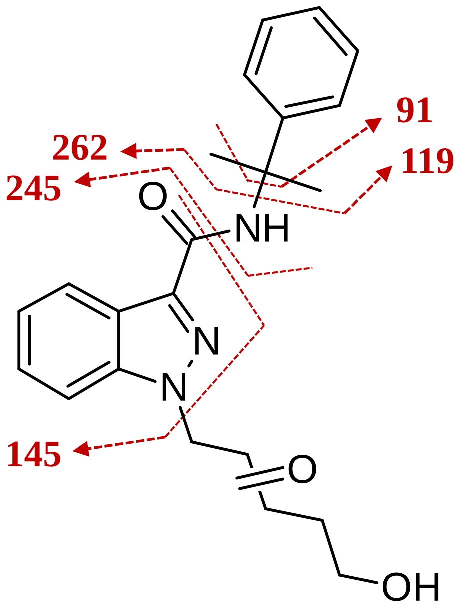

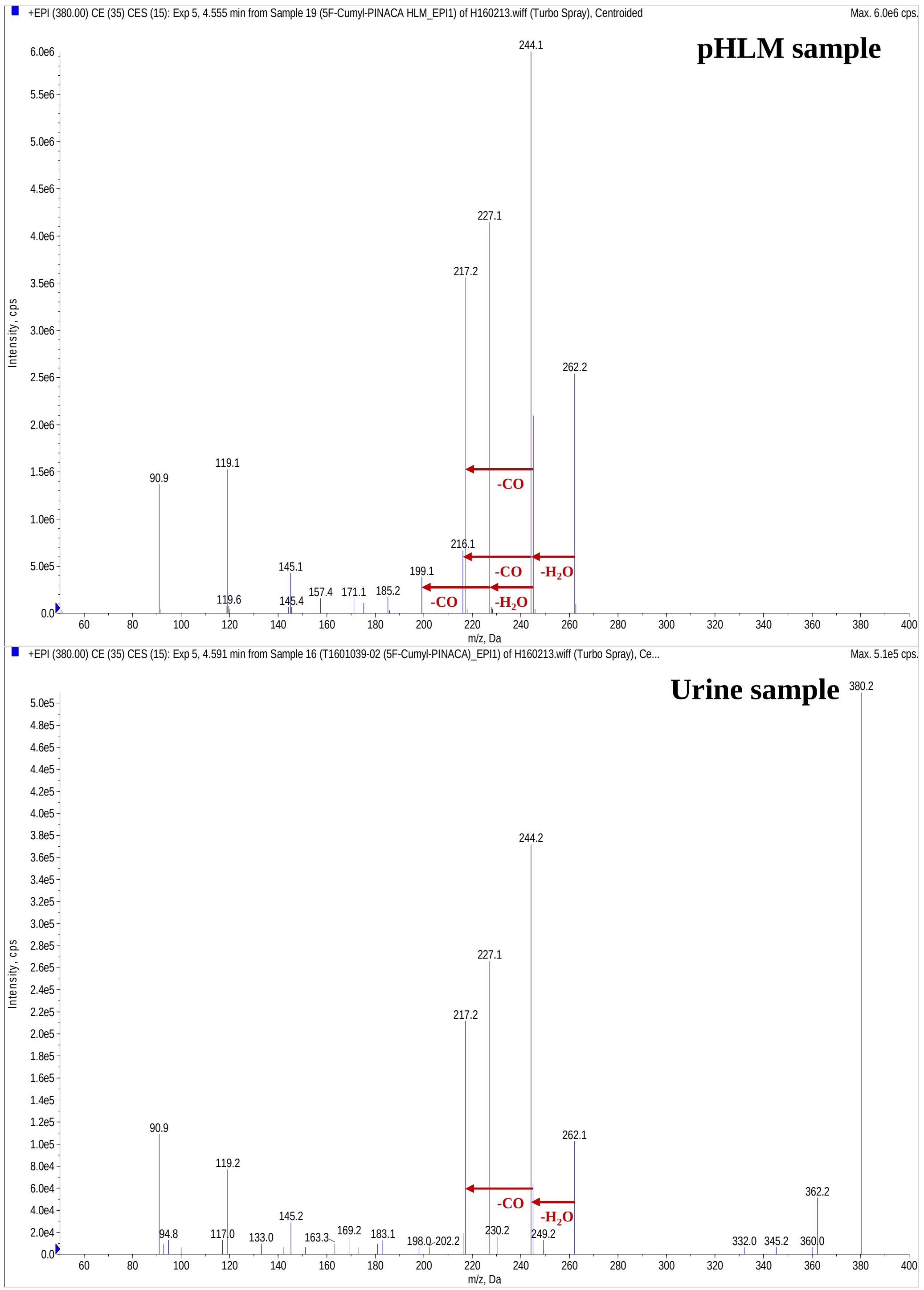

B02 (RT 4.5 min)

Molecular Formula:	$C_{22}H_{25}N_3O_3$
Monoisotopic Mass:	379.1896 Da
$[M+H]^+$:	380.1969 Da

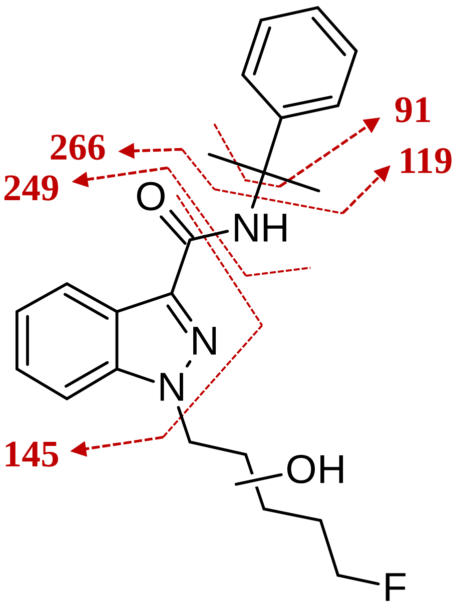

B03 (RT 4.7 min)

Molecular Formula: $C_{22}H_{26}FN_3O_2$
Monoisotopic Mass: 383.2009 Da
$[M+H]^+$: 384.2082 Da

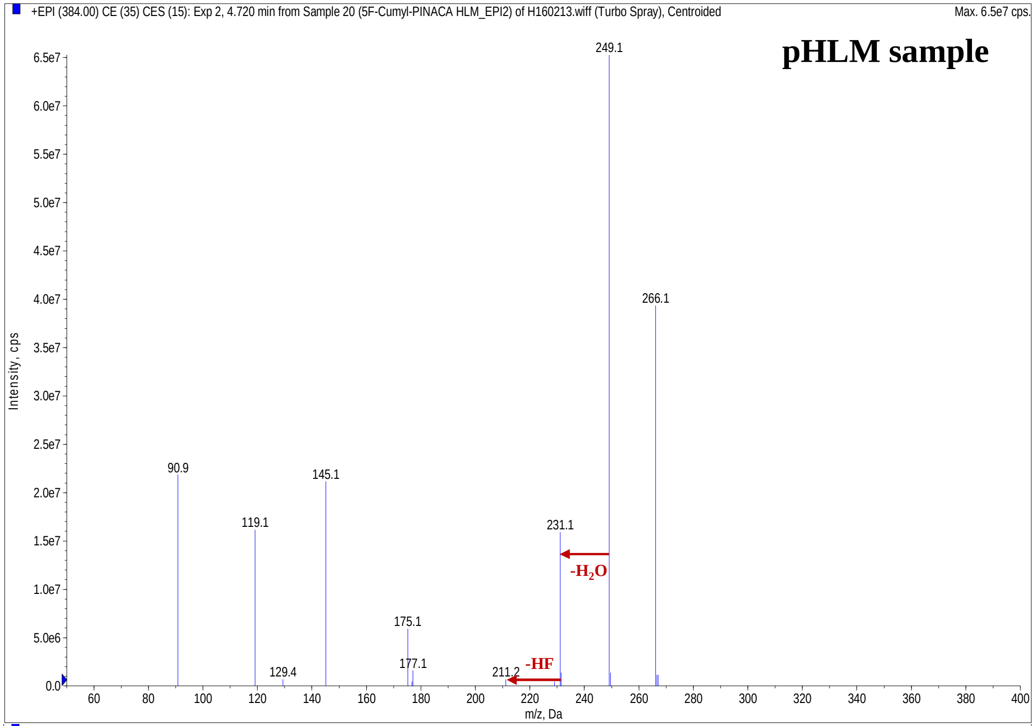

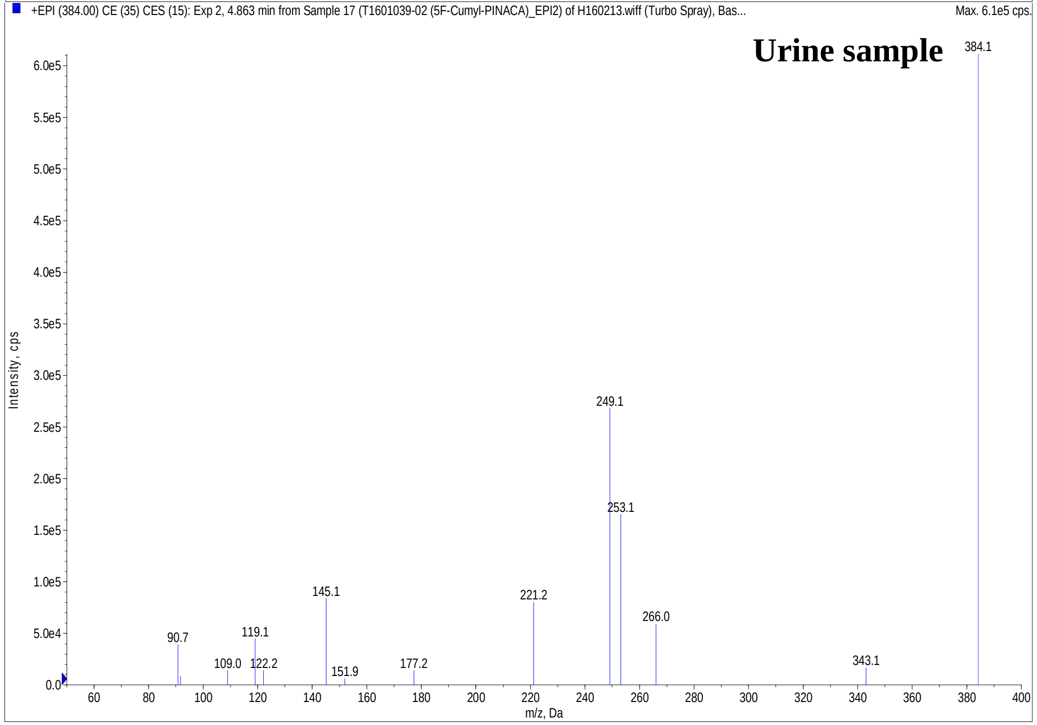

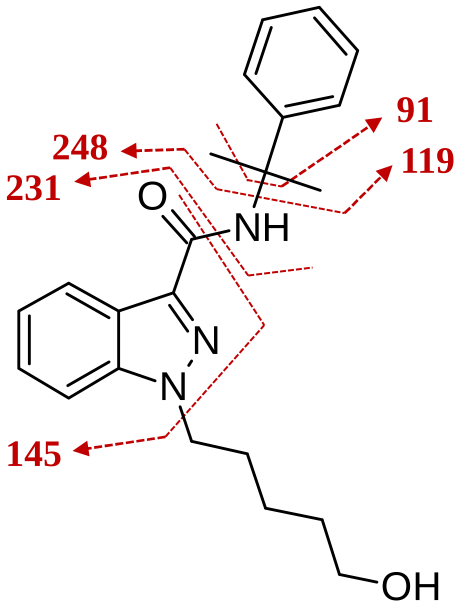

B04 (RT 4.8 min)

Molecular Formula: $C_{22}H_{27}N_3O_2$
Monoisotopic Mass: 365.2103 Da
$[M+H]^+$: 366.2176 Da

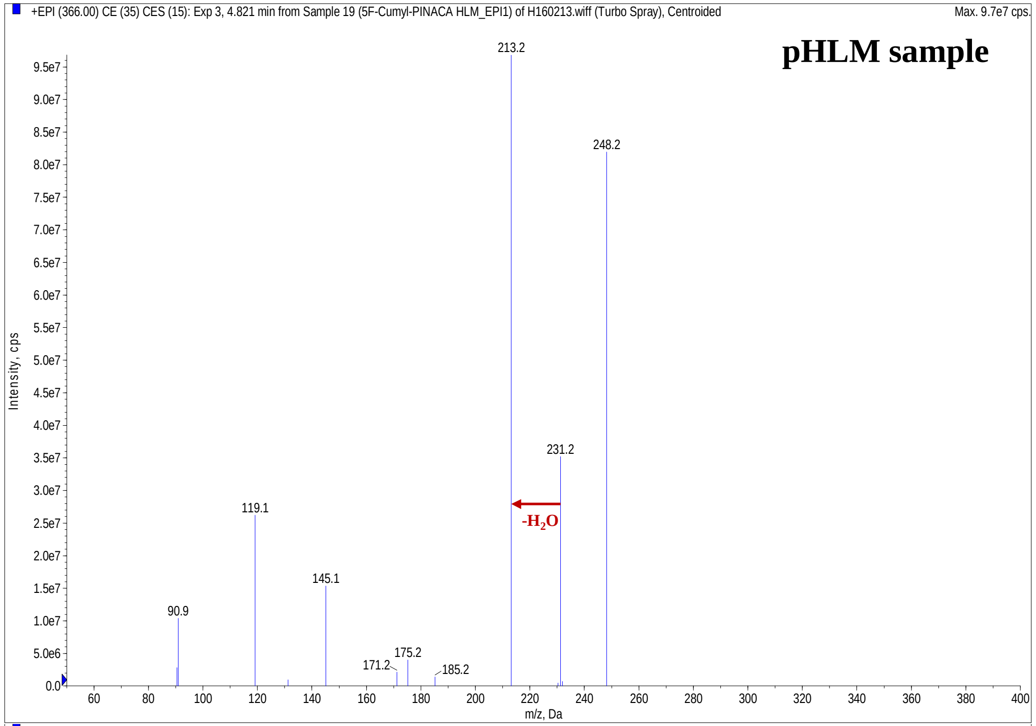

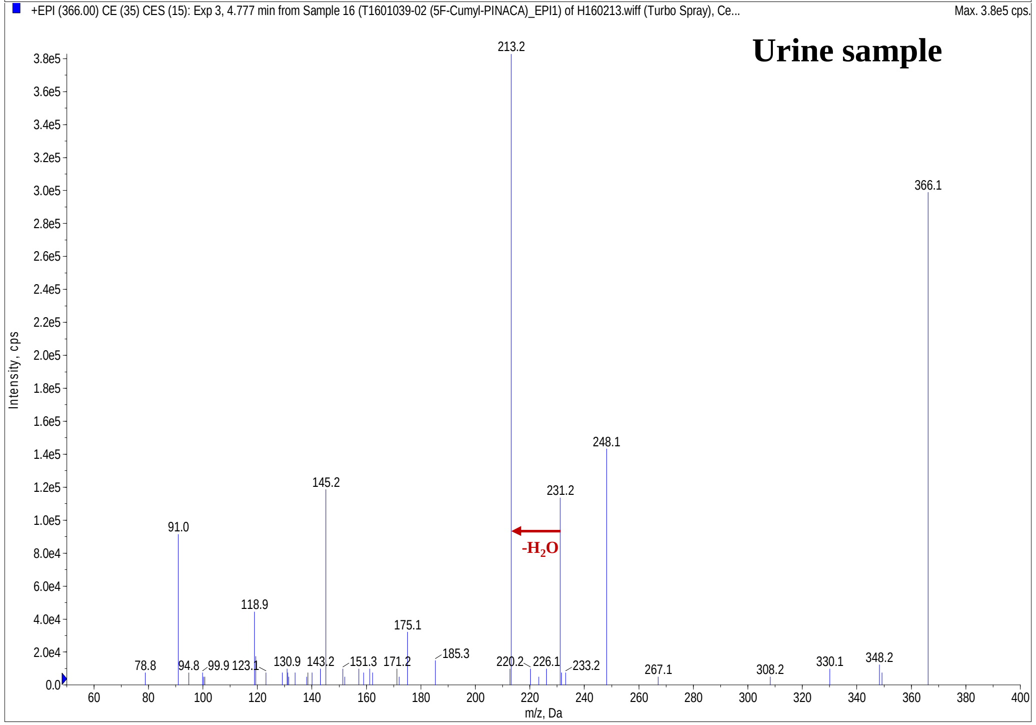

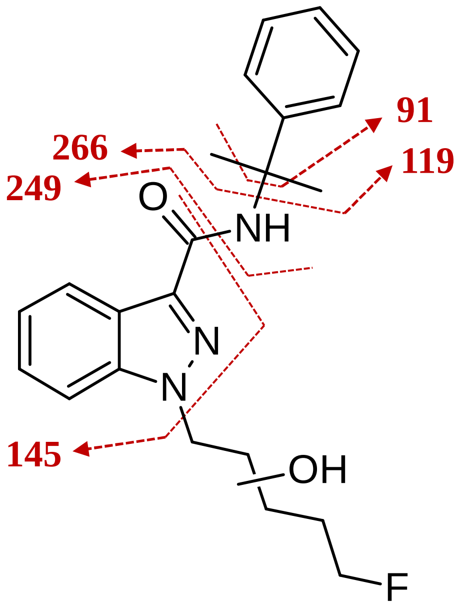

B05 (RT 4.8 min)

Molecular Formula: $C_{22}H_{26}FN_3O_2$
Monoisotopic Mass: 383.2009 Da
$[M+H]^+$: 384.2082 Da

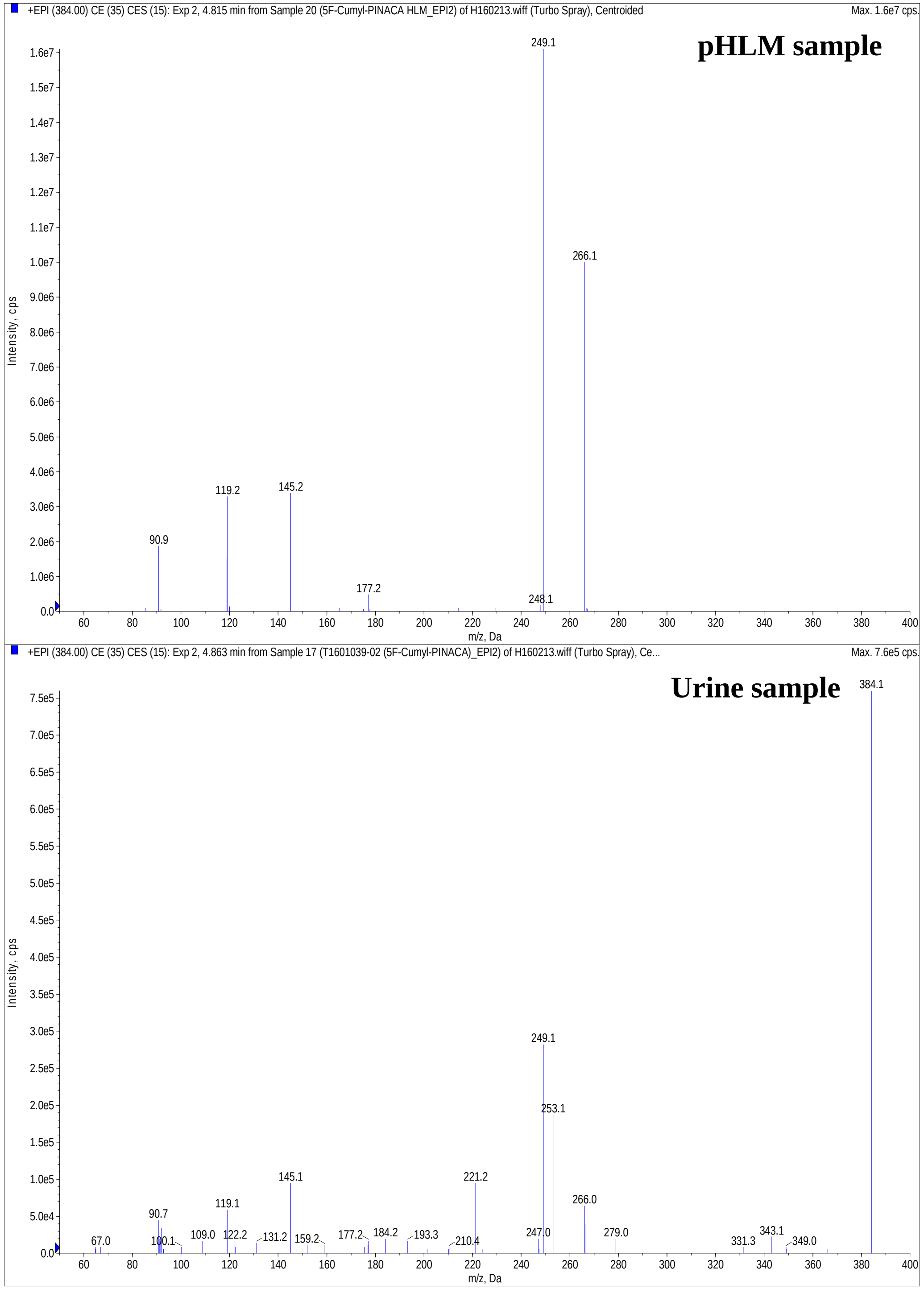

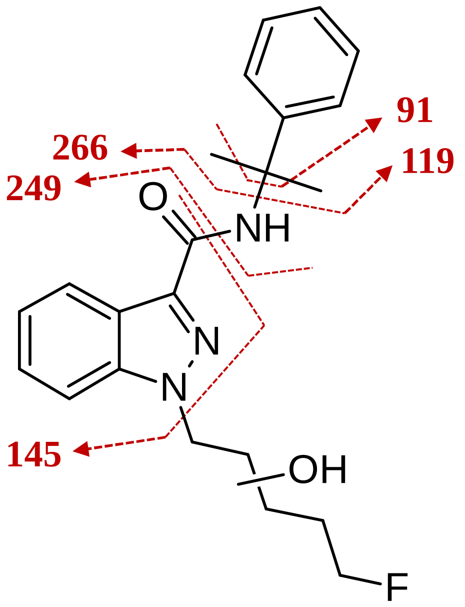

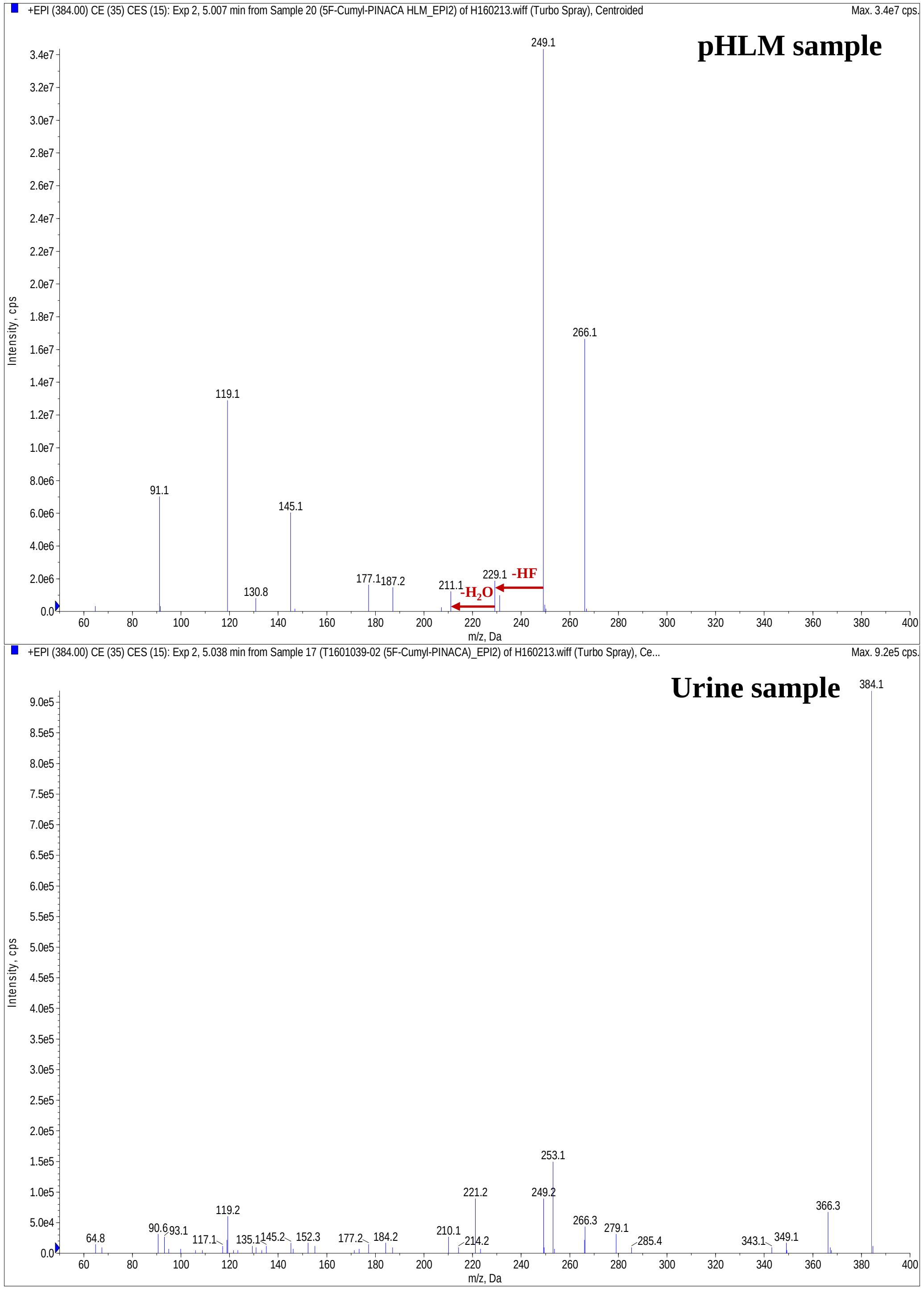

B06 (RT 5.0 min)

Molecular Formula:	$C_{22}H_{26}FN_3O_2$
Monoisotopic Mass:	383.2009 Da
$[M+H]^+$:	384.2082 Da
