# Supplementary material for: 5F-Cumyl-PINACA in ‘e-liquids’ for electronic cigarettes: comprehensive characterization of a new type of synthetic cannabinoid in a trendy product including investigations on the in vitro and in vivo phase I metabolism of 5F-Cumyl-PINACA and its non-fluorinated analog Cumyl-PINACA
Source: Forensic Toxicol. 2018 Nov 13;37(1):186–96. doi: 10.1007/s11419-018-0451-8 (PMC6315005; doi:10.1007/s11419-018-0451-8)
Supplement: Supplementary file 2 — Supplementary material 2 (PDF 322 kb) [file 11419_2018_451_MOESM2_ESM.pdf]

Electronic supplementary material – Forensic Toxicology

5F-Cumyl-PINACA in 'e-liquids' for electronic cigarettes: comprehensive characterization of a new type of synthetic cannabinoid in a trendy product including investigations on the in vitro and in vivo phase I metabolism of 5F-Cumyl-PINACA and its non-fluorinated analog Cumyl-PINACA

Verena Angerer[1,2,†], Florian Franz[1,2,3,†], Bjoern Moosmann[4], Philippe Bisel[5], Volker Auwärter[1,2,*]

[1] Institute of Forensic Medicine, Forensic Toxicology, Medical Center – University of Freiburg, Albertstr. 9, 79104 Freiburg, Germany

[2] Faculty of Medicine, University of Freiburg, Breisacher Str. 153, 79110 Freiburg, Germany

[3] Hermann Staudinger Graduate School, University of Freiburg, Hebelstr. 27, 79104 Freiburg, Germany

[4] Institute of Forensic Medicine, Forensic Toxicology, Kantonsspital St.Gallen, Rorschacher Str. 95, 9007 St.Gallen, Switzerland

[5] Institute for Pharmaceutical Sciences, University of Freiburg, Albertstr. 25, 79104 Freiburg, Germany

[†] These authors have contributed equally to this manuscript.

* Corresponding author:

Prof. Volker Auwärter
Tel.: +49 761 203 6862
Fax: +49 761 203 6826
Email: volker.auwaerter@uniklinik-freiburg.de

Table S1 Specific MRM mass transitions of Cumyl-PINACA and its detected metabolites

**Type**	**Q1**	**Q3**	**Dwell time [msec]**	**DP [V]**	**EP [V]**	**CE [V]**	**CXP [V]**	**Metabolite ID**
Cumyl-PINACA	350	215	20	40	8	30	25	A00
	350	145	20	40	8	55	17	
Mono-hydroxylation (+O)	366	231	20	40	8	30	25	A08, A09, A10
	366	145	20	40	8	55	17	
Di-hydroxylation (+2O)	382	231	20	40	8	30	25	A01
	382	145	20	40	8	55	17	
Di-hydroxylation (+2O)	382	247	20	40	8	30	25	A02, A03
	382	145	20	40	8	55	17	
Dihydrodiol (+2H, +2O)	384	249	20	40	8	30	25	A06
	384	119	20	40	8	55	17	
(+2O, -2H)	380	229	20	40	8	30	25	A04
	380	145	20	40	8	55	17	
(+2O, -2H)	380	245	20	40	8	30	25	A05, A07
	380	145	20	40	8	55	17	

Q;1 *m/z* of the precursor ion
Q3; *m/z* of the fragment ion
DP; Declustering potential
EP; Entrance potential
CE; Collision energy
CXP; Cell exit potential

Table S2 Specific MRM mass transitions of 5F-Cumyl-PINACA and its detected metabolites

**Type**	**Q1**	**Q3**	**Dwell time [msec]**	**DP [V]**	**EP [V]**	**CE [V]**	**CXP [V]**	**Metabolite ID**
5F-Cumyl-PINACA	368	233	10	65	12	30	8	B00
	368	145	10	65	12	55	5	
Monohydroxylation (+O)	384	249	10	65	12	30	8	B03, B05, B06
	384	145	10	65	12	55	5	
Dihydrodiol (+2H, +2O)	402	267	10	65	12	30	8	B01
	402	179	10	65	12	55	5	
Defluorination (+H, +O, -F)	366	231	10	65	12	30	8	B04
	366	145	10	65	12	55	5	
N-Pentanoic acid (+2O, -H, -F)	380	245	10	65	12	30	8	B02
	380	145	10	65	12	55	5	

Q1; *m/z* of the precursor ion
Q3; *m/z* of the fragment ion
DP; Declustering potential
EP; Entrance potential
CE; Collision energy
CXP; Cell exit potential

Table S3 Detected in vivo phase I metabolites of Cumyl-PINACA in each of the urine samples in the order of their retention time (RT)

ID	Type	RT [min]	Formula	$[M+H]^+$	Urine 00 0 h	Urine 01 0.2 h	Urine 02 3.2 h	Urine 03 6 h	Urine 04 16 h	Urine 05 20.3 h	Urine 06 22.3 h	Urine 07 26.3 h	Urine 08 29.3 h	Urine 09 37.2 h	Urine 10 39 h	Urine 11 88.5 h	Urine 12 110.3 h	Urine 13 134 h	Urine 14 157.5 h	Urine 15 182.5 h	Urine 16 205.5 h	pHLM
A01	Dihydroxylation	3.3	$C_{22}H_{27}N_3O_3$	382.2125	n.d.	n.d.	4.5E+04	6.4E+05	4.3E+05	2.0E+05	2.1E+05	2.0E+05	1.5E+05	1.1E+05	1.1E+05	n.d.	n.d.	n.d.	n.d.	n.d.	n.d.	1.9E+06
A02	Dihydroxylation	3.4	$C_{22}H_{27}N_3O_3$	382.2125	n.d.	n.d.	4.7E+04	1.2E+06	1.1E+06	3.9E+05	2.1E+05	1.8E+05	1.2E+05	6.7E+04	5.5E+04	n.d.	n.d.	n.d.	n.d.	n.d.	n.d.	2.8E+07
A03	Dihydroxylation	3.5	$C_{22}H_{27}N_3O_3$	382.2125	n.d.	n.d.	1.1E+05	3.1E+06	3.9E+06	1.6E+06	1.3E+06	1.4E+06	1.0E+06	6.0E+05	5.8E+05	n.d.	n.d.	n.d.	n.d.	n.d.	n.d.	4.2E+07
A04	(+2O, -2H)	3.7	$C_{22}H_{25}N_3O_3$	380.1969	n.d.	n.d.	5.4E+04	1.3E+06	9.8E+05	4.9E+05	6.7E+05	6.2E+05	5.2E+05	3.3E+05	3.6E+05	n.d.	n.d.	n.d.	n.d.	n.d.	n.d.	2.6E+06
A05	(+2O-, 2H)	4.2	$C_{22}H_{25}N_3O_3$	380.1969	n.d.	n.d.	1.7E+05	6.6E+06	8.8E+06	1.3E+06	2.3E+06	1.0E+06	1.4E+06	3.4E+05	5.8E+05	n.d.	n.d.	n.d.	n.d.	n.d.	n.d.	3.7E+07
A06	Dihydrodiol	4.3	$C_{22}H_{29}N_3O_3$	384.2282	n.d.	n.d.	5.0E+04	9.3E+05	2.2E+05	1.1E+05	1.5E+05	1.2E+05	9.1E+04	4.8E+04	4.1E+04	n.d.	n.d.	n.d.	n.d.	n.d.	n.d.	1.5E+06
A07	(+2O, -2H)	4.5	$C_{22}H_{25}N_3O_3$	380.1969	n.d.	n.d.	n.d.	8.4E+04	1.1E+05	5.2E+04	6.0E+04	4.2E+04	2.1E+04	1.7E+04	n.d.	n.d.	n.d.	n.d.	n.d.	n.d.	n.d.	2.9E+06
A08	Monohydroxylation	4.8	$C_{22}H_{27}N_3O_2$	366.2176	n.d.	n.d.	1.1E+06	1.7E+07	4.1E+06	1.4E+06	1.5E+06	7.7E+05	5.7E+05	4.5E+05	2.9E+05	2.6E+04	6.1E+04	2.7E+04	1.5E+04	1.3E+04	1.1E+04	7.3E+07
A09	Monohydroxylation	5.2	$C_{22}H_{27}N_3O_2$	366.2176	n.d.	n.d.	3.8E+05	7.6E+06	2.5E+06	9.8E+05	9.1E+05	6.1E+05	4.4E+05	3.9E+05	2.9E+05	2.9E+04	4.2E+04	1.6E+04	1.2E+04	n.d.	n.d.	4.6E+07
A10	Monohydroxylation	5.6	$C_{22}H_{27}N_3O_2$	366.2176	n.d.	n.d.	1.5E+05	2.9E+06	8.7E+05	3.4E+05	5.0E+05	3.6E+05	2.7E+05	2.0E+05	2.0E+05	2.1E+04	2.1E+04	7.0E+03	3.6E+03	n.d.	n.d.	1.1E+07

n.d.: Not detectable

The peak area of each metabolite was normalized to a creatinine value of 100 mg/dL

The time point of sampling is given in hours

Urine 00 was taken directly before oral drug intake

Urine samples 01 - 16 were taken after the self-administration

In vitro data after pHLM incubation of Cumyl-PINACA are given for comparison

Table S4 NMR data of the unknown compound detected in four e-liquids (for numbering see Fig. S3)

Carbon No.	^{1}H Δ (ppm)	Multiplicity	*J* (Hz)	^{13}C Δ (ppm)
1				161.8
3'				137.8
3'a				122.7
4'	8.33 (1 H)	td	8.38; 1.02	123.1
5'	7.20-7.25 (1 H)	m		122.4
6'	7.38-7.40 (1 H)	m		126.6
7'	7.38-7.40 (1 H)	m		108.9
7a'				140.8
1"	4.41 (2 H)	t	7.11	49
2"	2.01 (2 H)	quint.	7.35	29.3
3"	1.44-1.54 (2 H)	m		22.6
4"	1.68-1.83 (2 H)	m		29.9
5"	4.45 (2 H)	dt	5.88; 47.07	83.9 (d)
1"'				55.8
2"'	1.87 (6 H)	s		29.5
1""				147.3
2""+ 6""	7.50-7.55 (2 H)	m		124.7
3""+ 5""	7.32-7.38 (2 H)	m		128.3
4""	7.20-7.25 (1 H)	m		126.5

$CDCl_3$ was used as a solvent

Table S5 In vivo phase I metabolites of 5F-Cumyl-PINACA detected in authentic urine samples in the order of their retention time (RT)

ID	Type	RT [min]	Formula	$[M+H]^+$	pHLM [Peak area]	Urine [Peak area]
B01	Dihydrodiol	3.60	$C_{22}H_{28}FN_3O_3$	402.2187	7.0E+06	5.6E+04
B02	*N*-Pentanoic acid	4.50	$C_{22}H_{25}N_3O_3$	380.1969	9.3E+06	1.8E+05
B03	Monohydroxylation	4.70	$C_{22}H_{26}FN_3O_2$	384.2082	6.1E+07	3.9E+05
B04	Defluorination	4.80	$C_{22}H_{27}N_3O_2$	366.2176	6.0E+07	2.2E+05
B05	Monohydroxylation	4.80	$C_{22}H_{26}FN_3O_2$	384.2082	5.8E+07	2.7E+05
B06	Monohydroxylation	5.00	$C_{22}H_{26}FN_3O_2$	384.2082	4.4E+07	1.5E+05

In vitro data after pHLM incubation of 5F-Cumyl-PINACA are given for comparison
